# A review of patient-reported outcome measures to assess female infertility-related quality of life

**DOI:** 10.1186/s12955-017-0666-0

**Published:** 2017-04-27

**Authors:** Helen Kitchen, Natalie Aldhouse, Andrew Trigg, Roberto Palencia, Stephen Mitchell

**Affiliations:** 1 DRG Abacus, Manchester, UK; 2 Ferring Pharmaceuticals A/S, Copenhagen, Denmark; 3 DRG Abacus, Bicester, UK

**Keywords:** Female infertility, Quality of life, Patient-reported outcomes, Psychometric, Validation

## Abstract

**Background:**

Infertility has a negative impact on quality of life (QoL) and well-being of affected individuals and couples. A variety of patient-reported outcome (PRO) measures to assess infertility-related QoL are available; however, there is a concern regarding potential issues with their development methodology, validation and use. This review aimed to i) identify PRO measures used in infertility interventional studies ii) assess validation evidence to identify a reliable, valid PRO measure to assess changes in QoL or treatment satisfaction in clinical studies with female patients following treatment with novel therapies iii) identify potential gaps in evidence for validity.

**Methods:**

A structured literature search of Medline, Embase, and the Cochrane Library (accessed in September 2015) was conducted using pre-defined search terms. The identified publications were reviewed applying eligibility criteria to select interventional female infertility studies using PROs. Infertility-specific PRO measures assessing QoL, treatment satisfaction or psychiatric health, and included in studies by ≥2 research groups were selected and critically reviewed in light of scientific and regulatory guidance (e.g. FDA PRO Guidance for Industry) for evidence of content validity, psychometric strength, and patient acceptability.

**Results:**

The literature search and hand-searching yielded 122 publications; 78 unique PRO measures assessing QoL, treatment satisfaction or psychiatric health were identified. Five PRO measures met the selection criteria for detailed review: Fertility Quality of Life (FertiQoL); Fertility Problem Inventory (FPI); Fertility Problem Stress (FPS); Infertility Questionnaire (IFQ); Illness Cognitions Questionnaire adapted for Infertility (ICQ-I). None of the PRO measures met all validation criteria. The FertiQoL was the most widely used infertility-specific PRO measure to assess QoL in interventional studies, with reasonable evidence for adequate content validity, psychometric strength, and linguistic validation. However, gaps in evidence remain including test-retest reliability and thresholds for interpreting clinically important changes. While the FPI demonstrated reasonable evidence for content and psychometric validity, its utility as an outcome measure is limited by a lack of recall period.

**Conclusion:**

The FertiQoL and the FPI are potentially useful measures of infertility-related QoL in interventional studies. Further research is recommended to address gaps in evidence and confirm both PRO measures as reliable assessments of patient outcomes.

**Electronic supplementary material:**

The online version of this article (doi:10.1186/s12955-017-0666-0) contains supplementary material, which is available to authorized users.

## Background

Infertility is defined by the World Health Organisation (WHO) as “a disease of the reproductive system defined by the failure to achieve a clinical pregnancy after 12 months or more of regular unprotected sexual intercourse”[1], and is estimated to affect as many as 48.5 million couples worldwide [2]. Female infertility can be caused by many different factors including age which is a major determining factor [3], physiological dysfunction (this is also a factor in male, or couples infertility) [4], lifestyle (e.g. obesity, low body weight, smoking), and other unidentified causes [3]. Increasing evidence suggests that infertility represents a significant psychological burden to the affected individuals or couples as it can have a negative impact on their quality of life (QoL) and psychological and social well-being [5–9]. Furthermore, evidence suggests that women may be more substantially affected by infertility than their partners with respect to mental health, social functioning, and emotional behaviour [5, 10, 11].

Current infertility treatments such as surgical measures to treat genital tract obstruction or endometriosis, or hormone treatments to restore ovulatory function in women are aimed to reverse the primary causes of infertility; however, in cases where initial treatments are not successful or infertility is unexplained, assisted reproductive technology (ART), which includes in vitro fertilisation (IVF) and intracytoplasmic sperm injection (ICSI), is the treatment of choice [3]. While infertility treatments are successful in a considerable proportion of cases [12], they often have a negative impact on the patients’ QoL. Hormone treatments may have various psychological side-effects and IVF treatment can be invasive, time consuming and stressful, further contributing to the overall burden of infertility [13, 14]. Thus, assessing the effects of novel infertility therapies on QoL from a patient perspective is important and may lead to improved patient outcomes [15, 16].

The World Health Organisation defines QoL as “individuals’ perception of their position in life in the context of the culture and value systems in which they live and in relation to their goals, expectations, standards and concerns”[17]. It is a broad ranging concept affected in a complex way by the person’s physical health, psychological state, level of independence, social relationships, personal beliefs and their relationship to salient features in the environment [17].

Quality of life can be assessed by using generic or disease-specific measures, with the latter being preferable as they include questions which focus on specific aspects of the condition [18]. The use of patient-reported outcome (PRO) measures specifically designed for the assessments of infertility-related QoL has gradually increased in the last decade [18], possibly as a result of clear regulatory standards which PRO measures employed in clinical trials must meet, and a recognised need to understand and assess patient’s wellbeing in clinical practice [19–22]. Despite the availability of a variety of measures designed to assess infertility-related QoL of individuals or couples, there is still a concern regarding potential issues with the development methodology, validation and use of the existing PRO measures of infertility-related QoL [5, 15]. Given the importance of accurately assessing patient outcomes in relation to treatment and treatment satisfaction from a patient perspective, there is a need to identify robust, reliable, and validated PRO measures for use in clinical studies for the assessment of new infertility treatments.

The purpose of this research was therefore to identify reliable and valid PRO measures to assess changes in QoL or treatment satisfaction in clinical studies with female patients following treatment with novel therapies. The objectives of this structured literature review were to i) identify PRO disease-specific measures used in female infertility interventional studies ii) understand how PRO measures are used in infertility interventional studies, iii) identify and review the evidence that supports the validation of each PRO measure, and iv) identify any potential gaps in evidence for validity.

## Methods

This research was conducted in two stages: i) identification of existing patient-reported outcome (PRO) measures used in female infertility interventional studies via a structured literature review; ii) detailed assessment of the most relevant PRO measures, according to specified selection criteria, for content validity (defined as the extent to which the instrument measures concepts of interest such as QoL, psychiatric health, treatment satisfaction [22]), psychometric performance (defined as an assessment of the measurement properties of the PRO measure [22]) and practical considerations (i.e. cross-cultural feasibility and burden to patient) on their usage in the intended population.

### Identification of patient-reported outcome measures via structured literature review

The present structured literature review was conducted following a robust and reproducible methodology for the identification of relevant publications; a full protocol (not registered in a publicly available database) for the literature review was developed and executed to answer the objectives of this review.

The inclusion criteria were restricted to studies using disease-specific or generic PRO measures to determine the QoL of individuals affected by female factor infertility prior to, or while receiving infertility treatment. No restrictions were applied on type of intervention, comparator, country, language and date of publication of the studies. Further details about the eligibility criteria for study inclusion are available in the online supporting information (see Additional file 1: Table S1).

To identify relevant studies, the following databases were searched on 15^th^ September 2015: MEDLINE (including MEDLINE in process, from 1946); Embase (1980–2015); The Cochrane Library (Evidence based medicine reviews in OVID) including: Cochrane Central Register of Controlled Trials August 2015, Cochrane Database of Systematic Reviews 2005 to August 2015, Database of Abstracts of Reviews of Effects 2^nd^ Quarter 2015, Health Technology Assessment 3^rd^ Quarter 2015, NHS Economic Evaluation Database 2^nd^ Quarter 2015. A detailed search strategy was developed to identify all relevant studies from the published literature; details on the full search strategies used are provided in the online supporting information (see Additional file 2: Tables S2–S4).

The following additional sources were hand-searched: reference lists of included studies; relevant systematic reviews and meta-analyses identified in the electronic database searches and initially excluded; conference proceedings (American Society for Reproductive Medicine [ASRM]; European Society for Human Reproduction and Embryology [ESHRE]; International Society For Pharmaco-economics and Outcomes Research [ISPOR]; International Society for Quality of Life Research [ISOQOL]); clinical trial registries (ClinicalTrials.gov on 28^th^ September 2015); PRO databases (Patient-Reported Outcome and Quality of Life Instruments Database [PROQOLID]; Patient-Reported Outcome Labels Database [PROLabels] on 24^th^ September 2015).

Titles and abstracts of the studies identified in the database searches were screened by one experienced analyst (NA), applying the eligibility criteria for study inclusion (provided in the online supporting information, Table S1), and non-relevant studies were excluded (first pass). Studies that could not be excluded on the basis of title and abstract were retrieved for full publication review (second pass) by all members of the study team. Studies, abstracts, clinical trials and PRO measures identified from hand-searches were assessed applying the eligibility criteria, and the relevant studies were included in the review.

### Detailed review of identified patient-reported outcome measures

The PRO measures reported in the included studies were extracted to form a list. The identified PRO measures were then shortlisted according to the selection criteria outlined in Table 1, to identify the measures deemed most appropriate for use in infertility interventional studies.

**Table 1:** Criteria for the selection of PRO measures for detailed review and psychometric evaluation

Criterion	Include	Exclude
Setting	• Used in an infertility treatment setting	• Used to assess the QoL/psychological health of people living with female infertility but not receiving treatment
Intended population/context of use	• Designed specifically to assess the QoL/treatment satisfaction/psychological health of individuals affected by female infertility	• It is a general QoL/treatment satisfaction/psychological health assessment tool that can be used in multiple disease areas
Treatment type	• It is not treatment-specific	• It is treatment-specific
Previous use in the literature	• Has been used by at least two separate research groups^a^	• Used in multiple publications but by a singular research group
Language	• Available in an English-language version	• Not available in an English-language version
Psychometric data	• Psychometric assessment of the PRO conducted, published in the literature, and accessible for use	• Psychometric assessment not conducted • Psychometric assessment conducted but results are not accessible in the literature

*PRO* patient reported outcome, *QoL* quality of life

^a^The authors of the publications of at least two studies using the PRO do not overlap

These short-listed PRO measures were then reviewed in light of the FDA PRO Guidance for Industry, which summarises best practice for PRO measures used in clinical development [22]. In particular, the selected PRO measures were assessed for content validity and psychometric performance in the intended population of use, and practical considerations for use in multi-national clinical research.

To conduct this evaluation, the development publication and any subsequent publications further assessing the psychometric properties of each PRO measure were retrieved via hand-searching.

Further details on the specific parameters considered for the assessment of the selected PRO measures are provided in the online supporting information (see Additional file 3: Tables S5–S7). In addition, a gap analysis to evaluate the evidence for content and psychometric validity of the selected PRO measures was also conducted, in context of FDA and EMA regulatory requirements [22, 23].

## Results

### Study selection

**Fig. 1:**
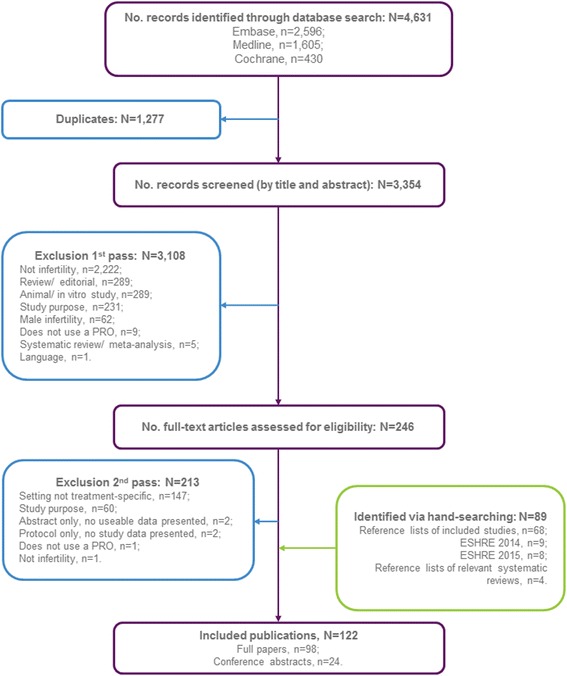
Literature review – flow diagram for study inclusion

The searching strategy (Fig. 1) identified a total of 4,631 citations. Following removal of duplicate citation records, 3,354 publications were screened by title and abstract yielding 246 potentially relevant publications which were screened on the basis of the full publication. Upon review of the full publications, a further 213 publications were excluded yielding 33 relevant publications. A list of excluded studies at second pass, along with the rationale for exclusion, is provided in the online supporting information (see Additional file 4: Table S8). Through hand-searching of additional publications, conference proceedings, clinical trials registries and PRO measure databases, an additional 89 relevant publications were identified. In total, 122 publications (98 full publications, 24 abstracts) reporting data on 115 unique studies were included in the review.

### Patient-reported outcome measures selected for detailed review

**Fig. 2:**
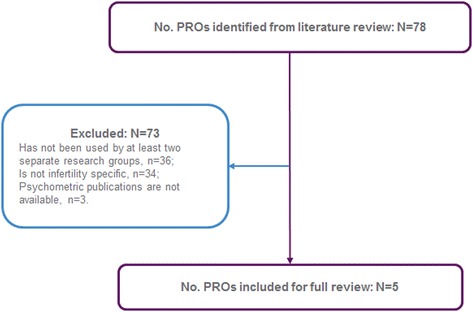
Flow diagram of PRO measures identified and selected for detailed review

**Fig. 3:**
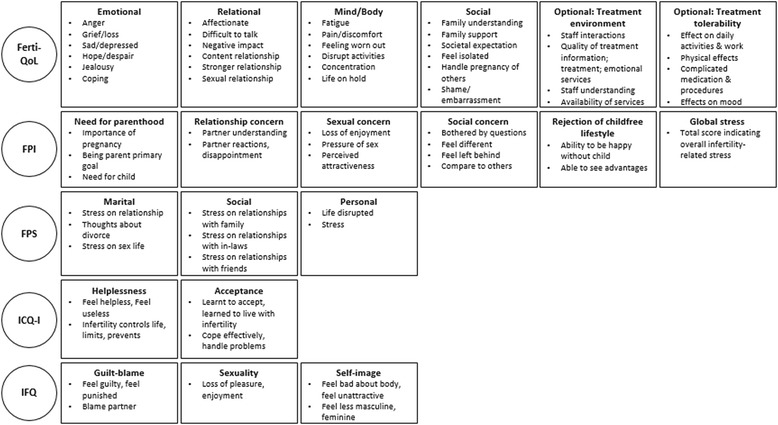
Overview of PRO domains and example items

The process followed to select the PRO measures for detailed review is described in Fig. 2. The 115 unique studies, identified through structured literature search, used a total of 78 unique PRO measures. Of these 78 unique PRO measures, five PRO measures, reported across 23 studies [24–48], were found to meet all selection criteria for detailed review and psychometric evaluation (Table 2). A summary of domains and example items included in each PRO measure is presented in Fig. 3. A list of the identified PRO measures excluded from further analysis, along with the reason for their exclusion, is provided in the online supporting information (see Additional file 5: Table S9).

**Table 2:** PRO measures identified for review and psychometric assessment

PRO Acronym	PRO measure name	Construct Assessed	Studies using PRO, n	Reference
FertiQoL	FertiQoL	Infertility-related quality of life Treatment satisfaction	12	[24, 27, 30, 32–34, 36, 38, 39, 43, 45, 47, 49]
FPI	Fertility Problem Inventory	Infertility-related stress	4	[35, 37, 41, 42]
IFQ	Infertility Questionnaire	Infertility-related emotional impairment	3	[28, 29, 40, 46]
FPS	Fertility Problem Stress	Infertility-related stress	2	[25, 44]
ICQ-I	Illness Cognitions Questionnaire – Adapted for infertility	Infertility-related feelings of helplessness and acceptance	2	[26, 48]

Study results may be reported in more than one publication, resulting in a number of references greater than the number of studies

*PRO* patient reported outcome

### Detailed review of the most relevant patient-reported measures for infertility-related QoL

The following five PRO measures of interest were reviewed: Fertility Quality of Life (FertiQoL); Fertility Problem Inventory (FPI); Fertility Problem Stress (FPS); Infertility Questionnaire (IFQ); Illness Cognitions Questionnaire-Adapted for infertility (ICQ-I). The validity of the PRO measures was assessed by considering a variety of characteristics, and therefore it was not possible to define a PRO measure as ‘valid’ or ‘not valid’. However, we were able to weigh the balance according to the findings of our detailed review. Results of statistical tests used to evaluate psychometric properties were interpreted as detailed in Table 3. An overview of findings for content and psychometric validity is presented in Table 4 and Table 5; practical considerations are summarised in Table 6.

**Table 3:** Interpretation of statistical tests

Property	Statistical test	Value	Interpretation
Internal consistency reliability [65]	Cronbach’s α	>0.70	Acceptable
Test-retest reliability [66]	Intraclass Correlation Coefficient	>0.75	Good reliability
Concurrent/convergent validity [63]	Spearman’s rank correlation coefficient	*r* ≤ 0.25 *r* = 0.26–0.49 *r* = 0.50–0.69 *r* = 0.70–0.89 *r* ≥ 0.90	Little if any correlation Low correlation Moderate correlation High correlation Very high correlation

**Table 4:** Overview of instrument content validity

Instrument name	Number of items	Acceptable respondent burden	Clarity of instructions	Clarity of item wording	Appropriate and balanced response options	Appropriate recall period
FertiQoL	36	✓	✓	✓	✓	X
FPI	46	✓	✓	X	X	X
FPS	14	✓	✓	✓	✓	X
ICQ-I	12	✓	n/av	X	X	n/av
IFQ	21	✓	✓	X	✓	X

*FertiQoL* Fertility Quality of Life Questionnaire, *FPI* Fertility Problem Inventory, *FPS* Fertility Problem Stress, *ICQ-I*, Infertility Cognitions Questionnaire adapted for Infertility, *IFQ* Infertility Questionnaire, *n/av* not available

**Table 5:** Overview of psychometric evidence

Instrument name	Validity				Reliability		Responsiveness	Clinically important difference thresholds available
	Structural validity	Convergent validity	Concurrent validity	Known groups validity	Internal consistency reliability	Test-retest reliability		
FertiQoL	✓	✓	X	✓	✓	X	X	X
FPI	✓	✓	X	X	✓	✓	X	X
FPS	✓	X	X	X	✓	X	X	X
ICQ-I	X	X	X	X	✓	X	X	X
IFQ	X	✓	X	✓	✓	✓	X	X

*FPI* Fertility Problem Inventory, *FPS* Fertility Problem Stress, *ICC* Intra-class Correlation Coefficient, *ICQ-I* Infertility Cognitions Questionnaire adapted for Infertility, *IFQ* Infertility Questionnaire

**Table 6:** Overview of practical considerations

Instrument name	Number of Language versions	Linguistic validation conducted	Length of measure (burden)	Availability of electronic version
FertiQoL	>30	✓	✓ 36 items	✓
FPI	>11	✓	X 46 items	X
FPS	2	✓	✓ 14 items	X
ICQ-I	3	X	✓ 12 items	X
IFQ	2	✓	✓ 20 items	X

*FertiQoL* Fertility Quality of Life Questionnaire, *FPI* Fertility Problem Inventory, *FPS* Fertility Problem Stress, *ICQ-I* Infertility Cognitions Questionnaire adapted for Infertility, *IFQ* Infertility Questionnaire, *ePRO* electronic patient-reported outcome

#### Fertility quality of life

The Fertility Quality of Life (FertiQoL) was developed in 2011 as an international instrument to measure QoL in men and women experiencing fertility problems and includes an additional module for the assessment of treatment satisfaction [15]. Of the infertility-specific PRO measures identified in this literature review, the FertiQoL was found to be the most widely used measure to assess QoL in interventional infertility studies, being used in 12 of the 23 identified studies [24, 27, 30, 32–34, 36, 38, 39, 43, 45, 47, 49]. The extensive cognitive debriefing in patients applied in the development of the FertiQoL suggests an adequate face and content validity in terms of number of items included (*n* = 36), respondent’s burden, clarity of instructions and balance in response options [15]. However, we found a lack of clarity in the recall period, which may prevent a consistent interpretation, and some potential complexity in item wording. In terms of psychometric strength, we found strong evidence for internal consistency reliability, as measured by Cronbach’s alpha values (α values range: 0.72–0.92) [15, 24, 50, 51], and construct validity – especially to support the structure of the conceptual framework of the FertiQoL [15, 51]. The convergent validity of the FertiQoL has been assessed in five studies validating various language versions of the FertiQoL [24, 50–53]. In these studies, moderate to low correlations were observed between the FertiQoL Core score and relevant scales of the Medical Outcomes Study Short Form 36 (SF-36) (correlation coefficients range: 0.32-0.53, *p* < 0.05) and Hospital Anxiety and Depression Scale (HADS) (correlation coefficients range: -0.65 to -0.67, *p* < 0.01), suggesting that the FertiQoL Core scale measures constructs related to mental health, as expected. Known groups validity has been assessed in four international validation studies; FertiQoL scores were found to differ significantly between patients grouped according to clinically significant treatment outcomes (e.g. no pregnancy, pregnancy and treatment cancellation) [24, 50–52]. We found no evidence for test-retest reliability (stability over time), and a lack of established clinically important differences (CIDs).

#### Fertility problem inventory

The Fertility Problem Inventory (FPI) was developed in 1999 as a specific measure of infertility-related stress in males and females [54] and has been utilised in a number of interventional studies [35, 37, 41, 42]. The FPI was developed through a qualitative literature review to inform the underlying concepts [54]. Limited evidence was found for cognitive testing with patients to fully establish content validity of the FPI. However, while content validity was considered adequate in terms of number of items included (*n* = 46), conceptual framework [55], respondent’s burden and clarity of instructions, the response scale options may not be balanced due to the lack of a true midpoint of the scale (neutral response). Furthermore, we found some inconsistency in the item wording, which may have an impact on the validity of the data, and a lack of clarity in the recall period which may limit the use of the FPI measure at multiple time points during a clinical trial. With regard to psychometric strength, evidence suggests good internal consistency reliability for the FPI scales (as measured by Cronbach’s alpha values range: 0.77–0.93) [31, 54, 56–59], test-retest reliability (correlation coefficient 0.83 following a 30-day interval) [54], and an adequate construct validity, which was assessed by exploring inter-correlations between each domain scale (correlation coefficients range: 0.26–0.66, all *p* < 0.05) [54]. Furthermore, convergent validity was demonstrated by a significant correlation between the FPI and some theoretically related measures such as the Beck Depression Inventory (correlation coefficients range: 0.29-0.62, all *p* < 0.05), State Trait Anxiety Index (correlation coefficients: 0.16–0.37, all *p* < 0.05) and Dyadic Adjustment Scale (marital adjustment, correlation coefficients range: -0.14 to -0.40, all *p* < 0.01) [54].

#### Fertility problem stress

The Fertility Problem Stress (FPS) was originally developed in 1991 to assess infertility-related stress [25] and more recently updated to include further input from patients through item-testing [44, 60]. Adequate content validity was observed for the FPS based on a clear response scales and item wording, both validated by patient involvement in the development process, and a low completion burden (14 items included) [44, 60]. Although no conceptual framework is available for the FPS, factor analysis has confirmed its three domain structure [61]. However, the lack of clear recall period may affect reliability of patient responses when administered longitudinally.

With regard to psychometric strength, evidence for internal consistency reliability is available in the form of Cronbach’s alpha values (α values ranges: women, 0.73–0.81; men, 0.72–0.84) which suggest acceptable internal consistency and reliability in measuring a well-defined construct for all FPS scores (38). Test-retest reliability has not been assessed for the FPS; it is therefore unclear whether scores would remain stable over time for patients experiencing no change in QoL, and confirmation that the measure can assess true change in relation to treatment. Convergent, divergent and inter-scale validity and known groups’ analysis have not been assessed for the FPS; however, confirmatory factor analysis has supported the structure of the measure (38). Responsiveness of the FPS has not been published and no CIDs in scores have been established.

#### Infertility questionnaire

The Infertility Questionnaire (IFQ) was developed in 1985 as a simple method of assessing the emotional impairment that accompanies infertility [62]. Unlike the FertiQoL, the IFQ does not assess treatment satisfaction, and has not been widely used in interventional studies, being used in three [28, 29, 40, 46] of the 115 identified studies. We found no published evidence for qualitative exploration of the IFQ to determine conceptual relevance and understanding. The IFQ was found to be adequate in terms of patient burden (21 items included) and clarity of instructions; however, item wording alternates between negatively and positively framed questions, potentially increasing the otherwise low patient burden [62]. The 4-point Likert response scale is skewed towards positive responses which may potentially create bias and/or ceiling effects. The vague recall period observed for the IFQ may also affect reliability of patient responses. With regard to psychometric strength, an acceptable internal consistency reliability, as measured by Cronbach’s alpha values (range: 0.72-0.83), together with an adequate test-retest reliability indicate that the IFQ is a stable assessment over time [62]. We found no evidence for assessment of structural validity, making it unclear whether the structure of the questionnaire is appropriate, and no evidence for known group validity. However, convergent validity, assessed via comparison between the IFQ and the Symptom Checklist 90 (SCL-90), was shown by a low to moderate correlation between the IFQ and the SCL-90 (correlation coefficient for the total test mean scores: 0.58), suggesting that the IFQ measures constructs related to psychiatric health, as expected [63]. No CIDs have been established for the IFQ.

#### Illness cognitions questionnaire-adapted for infertility

The Illness Cognitions Questionnaire-adapted for infertility (ICQ-I) was created in 2005 [48] as an adaptation of the ICQ, a generic assessment of cognition related to helplessness and acceptance in individuals with chronic diseases [64]. The ICQ-I was utilised in two interventional studies [26, 48]. As the ICQ-I was originally a generic measure, patients with a variety of chronic diseases were involved in the qualitative exploration to determine conceptual relevance and understanding of this measure [64]. Consequently, there is a lack of established evidence for content validity in an infertility patient population.

No conceptual framework is available for the ICQ, and although factor analysis has confirmed the structure of the measure in patients with rheumatoid arthritis and multiple sclerosis, no analysis has been conducted in an infertility population [64].

Internal consistency reliability was assessed in the initial psychometric validation of the original (non-disease specific) ICQ, and again when first adapted for infertility; Cronbach values suggest each domain is reliably measuring a well-defined construct (α values range: 0.86–089). Test-retest reliability was confirmed in the original version of the ICQ [64], but no test-retest reliability has been assessed in patients with infertility. Construct validity, confirmatory factor analysis and known groups’ analysis were assessed for the original ICQ measure, but not confirmed in an infertility population. Furthermore, responsiveness has not been assessed for the ICQ-I, and no CIDs in scores have been established.

## Discussion

The aim of this review was to identify reliable and valid PRO measures to assess changes in QoL or treatment satisfaction in clinical studies with female patients following treatment with novel therapies. Through a structured literature review, followed by a selection process based on specified eligibility criteria, the following five infertility-specific PRO measures were identified and reviewed for content and psychometric strength in light of the FDA PRO Guidance for Industry [22]: Fertility Quality of Life (FertiQoL); Fertility Problem Inventory (FPI); Fertility Problem Stress (FPS); Infertility Questionnaire (IFQ); Illness Cognitions Questionnaire-Adapted for infertility (ICQ-I).

Results from the literature review showed that the FertiQoL is the most widely used disease-specific PRO measure to assess infertility-related QoL in interventional studies [24, 27, 30, 32–34, 36, 38, 39, 43, 45, 47, 49]. A critical review of the FertiQoL suggests that there is reasonable evidence for an adequate content validity and reliability, including cognitive testing with patients, as well as acceptable psychometric properties which ensure internal consistency and construct reliability for this PRO measure [15]. Of the five PRO measures reviewed, the FertiQoL is the only one including an additional module for the assessment of treatment satisfaction; this may make the FertiQoL a particularly useful PRO measure for inclusion in clinical studies on infertility treatments, as it would allow the assessment of this specific aspect from a patient perspective.

With regard to the other four PRO measures reviewed, the FPS and the IFQ, followed by the FPI, were found to show overall the strongest evidence for content validity, especially in terms of clarity of instructions and balanced response options. The assessment of content validity for the ICQ-I was limited by the lack of established evidence in an infertility patient population, as this measure was originally designed to be a generic measure for patients with a variety of chronic diseases. A lack of clarity in the recall period was found to be a common issue for all reviewed PRO measures including the FertiQoL; this may lead to inconsistent interpretation of the findings and limit the ability to detect changes over time. Reasonable evidence for psychometric strength was also found for the FPI, FPS, IFQ and ICQ-I in terms of internal consistency reliability and construct validity, although, the evidence for the ICQ-I was based on unrelated patient populations. Test-retest reliability was found to be adequate for three of the four PRO measures (FPI, IFQ, ICQ-I); however, limited evidence was found for responsiveness of the measures to change and no thresholds for interpreting changes scores through were identified. All the reviewed PRO measures are available in more than one language version, with the FertiQoL been translated in 30 languages and the FPI in more than ten languages. In contrast, the remaining three PRO measures are only available in two or three different language versions: English and Danish for the FPS; English and Chinese for the IFQ; Dutch, English and Hebrew for the ICQ-I. However, we found scarce or no evidence for adequacy of linguistic and cultural validation for all the PRO measures, therefore further translations and/or linguistic validation may be beneficial for the use of these PRO measures in multinational clinical trials.

Although the FertiQoL was found to be the most widely used PRO measure to assess QoL in interventional infertility studies, some gaps in the evidence for psychometric strength remain. In particular, we noted a lack of test-retest reliability to ensure that the FertiQoL is a stable assessment over the time. In addition, we observed a lack of established CID thresholds for determining meaningful score changes, which combined with a vague recall period, might limit the interpretability of the findings in a clinical trial setting. A similar gap in evidence for established CIDs was also observed for the other reviewed PRO measures.

Whilst evidence for content and psychometric validity of PRO measures is paramount for use in clinical studies and, indeed, clinical practice, there are also practical considerations which affect the feasibility of PRO use. In particular, for longitudinal research where a PRO is to be completed at multiple time points, patient burden is an important consideration in terms of the PRO (e.g. length and complexity) and the reality of patient experience. Assessment of QoL in a therapeutic area which is impacted by multiple situational and psychosocial factors related to conception, pregnancy, and neo- and post-natal experience is inherently complex and thus selection of PRO measures and interpretation of the results obtained must be considered in context of such factors.

This review was conducted through a structured literature search to identify a comprehensive body of literature; however some limitations to this review need to be acknowledged. Firstly, only articles and PRO measures available in English language were included for review; therefore, it is possible that relevant PROs available in non-English languages only may have not been captured and reviewed. Furthermore, the study inclusion parameters limited the scope of this review to PRO measures which had been previously included in interventional studies. Subsequently, instruments which are early in development and have not yet been included in interventional studies might not have been included in this review. Secondly, the 1^st^ pass of abstract screening was completed by one analyst only; at 2nd pass all members of the study team reviewed the list of included studies. As a consequence, some studies using the identified PROs may not have been identified; however, this limitation was mitigated by extensive hand-searching. Finally, this review was undertaken without a qualitative assessment of patient’s experience, either through a review of existing qualitative literature or via primary research with women affected by infertility. Therefore, it was not possible to fully evaluate the conceptual relevance (and content validity) of the PRO measures reviewed.

Despite these limitations, the main findings of this review are in line with those reported in a recent systematic review (SR) assessing questionnaires used to measure QoL of infertile couples [18]. In this SR, the FertiQoL and the FPI were found to be valid measures for the evaluation of infertility problems and its treatment effects, and infertility-related stress respectively; although more investigations on the validity of both measures for use in different cultures and nations was recommended [18]. In contrast with the findings of this review, the same SR found that the FertiQoL and the FPI are rarely used to measure infertility-related QoL in infertility studies; however, it should be noted that this SR assessed and compared both disease-specific and generic PRO measures, such as the Short Form (36) Health Survey (SF-36) [18].

## Conclusions

The FertiQoL and the FPI are potentially useful measures of infertility-related QoL in clinical development of novel therapies; however, gaps in evidence for the PRO measures reviewed still remain. To ensure these PRO measures are valid, reliable assessments of patient QoL over time, further research is required to establish the recall period of the questionnaires, to define CIDs to improve guidance in the interpretation of clinically important changes, and to make multiple language translations available for use in multinational trials.

## Additional files

Additional file 1: Eligibility criteria for study inclusion in the literature review. Table presenting literature review inclusion criteria. (DOCX 47 kb)

Additional file 2: Search strategies. Three tables presenting the three search strategies used during literature review. (DOCX 55 kb)

Additional file 3: Detailed review of PRO measures. Three tables presenting further methodological details of the PRO review. (DOCX 53 kb)

Additional file 4: Publications excluded at second pass (screened by full paper). Table listing each publication excluded during second pass of the literature review, detailing first author, title, citation, and the reason for exclusion. (DOCX 82 kb)

Additional file 5: PROs excluded from detailed analysis. Table listing each identified PRO excluded from detailed analysis, detailing each PRO acronym, PRO name, no. studies identified as using the PRO, and the reason for exclusion. (DOCX 55 kb)

## Abbreviations

ART: Assisted reproductive technology
ASRM: American society for reproductive medicine
CID: Clinically important difference
ESHRE: European society for human reproduction and embryology
FertiQoL: Fertility quality of life questionnaire
FPI: Fertility problem inventory
FPS: Fertility problem stress
HADS: Hospital anxiety and depression scale
ICQ-I: Illness Cognitions Questionnaire - Adapted for Infertility
ICSI: Intracytoplasmic sperm injection
IFQ: Infertility questionnaire
ISOQOL: International society for quality of life research
ISPOR: International society for pharmacoeconomics and outcomes research
IVF: In-vitro fertilisation
n/av: Not available
PRO: Patient reported outcome
PROQOLID: The Patient-reported outcome and quality of life instruments database
QoL: Quality of life
SF-36: Short form (36) health survey
SR: Systematic review
WHO: World health organisation
