# Supplementary material for: A review of patient-reported outcome measures to assess female infertility-related quality of life
Source: Health Qual Life Outcomes. 2017 Apr 27;15:86. doi: 10.1186/s12955-017-0666-0 (PMC5408488; doi:10.1186/s12955-017-0666-0)
Supplement: Supplementary file 1 — Eligibility criteria for study inclusion in the literature review. Table presenting literature review inclusion criteria. (DOCX 47 kb) [file 12955_2017_666_MOESM1_ESM.docx]

Table S1: Eligibility criteria for study inclusion in the literature review

| Criterion | Include | Exclude |
| --- | --- | --- |
| Population | - Studies enrolling any number of individuals affected by female-factor infertility | - Studies enrolling couples only affected by male- and/or mixed-factor infertility - Publications without a clear description of the analysed population |
| Intervention | No restrictions | No restrictions |
| Comparator | No restrictions | No restrictions |
| Outcomes | - Studies using PROs to determine the QoL of individuals affected by female infertility.   - PROs may be infertility-specific or designed for generic use in any disease area. | - Studies using PROs to assess factors not related to the QoL of individuals affected by female infertility, including but not limited to:   - Assessments of patient personality   - Infertility coping strategies |
| Study design | - Studies assessing the QoL of individuals affected by female infertility while receiving infertility treatment | - Studies not reporting a clear description of the time points measured - Studies measuring post-natal QoL or long‑term QoL following infertility treatment - Case reports |
| Countries | No restrictions | No restrictions |
| Language | No restrictions | No restrictions |
| Date of publication | No restrictions† | No restrictions† |

Abbreviations: PRO, patient-reported outcome; QoL, quality of life

†The Embase and Medline databases includes only studies from 1980 and 1946 onwards, respectively. However, studies from published prior to these dates will be included if sourced via Cochrane or hand-searching
