# Supplementary material for: A review of patient-reported outcome measures to assess female infertility-related quality of life
Source: Health Qual Life Outcomes. 2017 Apr 27;15:86. doi: 10.1186/s12955-017-0666-0 (PMC5408488; doi:10.1186/s12955-017-0666-0)
Supplement: Supplementary file 2 — Search strategies. Three tables presenting the three search strategies used during literature review. (DOCX 55 kb) [file 12955_2017_666_MOESM2_ESM.docx]

Search strategies

Table S2: Search strategy for Embase 1980 to 2015 Week 37, accessed 15-September-2015

| # | Searches | Results |
| --- | --- | --- |
| 1 | exp female infertility/ | 37121 |
| 2 | infertility/ or infertil*.mp. | 102899 |
| 3 | fertil*.mp. | 198165 |
| 4 | or/1-3 | 264294 |
| 5 | (EQ-5D or EQ5D or Euroqol).mp. | 9156 |
| 6 | (Health utilities index or HUI).mp. | 2434 |
| 7 | (time trade off or ("TTO" adj2 "time trade")).mp. | 1156 |
| 8 | standard gamble.mp. | 862 |
| 9 | (15D or 16D or 17D).mp. | 2919 |
| 10 | (medical outcomes survey or MOS).mp. | 10341 |
| 11 | (Quality of wellbeing index or QWB).mp. | 214 |
| 12 | FertiQoL.mp. | 44 |
| 13 | (Fertility problem Inventory or FpI).mp. | 1601 |
| 14 | (Hopkins Symptom checklist or SCL).mp. | 8886 |
| 15 | WHOQOL.mp. | 2393 |
| 16 | Enrich Inventory.mp. | 6 |
| 17 | Sickness impact profile.mp. | 2895 |
| 18 | (Sexual Functioning Questionnaire or SFQ).mp. | 274 |
| 19 | ("quality of well-being scale" or "quality of wellbeing scale").mp. | 189 |
| 20 | exp questionnaire/ or questionnaire*.mp. | 614185 |
| 21 | Short form.mp. | 33417 |
| 22 | Instrument*.mp. | 516311 |
| 23 | (Patient reported outcome* or PRO).mp. | 197162 |
| 24 | PROM.mp. | 2375 |
| 25 | scale*.mp. | 770669 |
| 26 | or/5-25 | 1911677 |
| 27 | (QoL or HRQoL or HRQL).mp. | 56911 |
| 28 | exp "quality of life"/ or quality of life.mp | 366155 |
| 29 | Treatment satisfaction.mp. | 3071 |
| 30 | or/ 27-29 | 371551 |
| 31 | 4 and 26 and 30 | 830 |
| 32 | exp reproducibility/ or reproducib*.mp. | 254866 |
| 33 | reliab*.mp. | 435248 |
| 34 | valid*.mp. | 651386 |
| 35 | responsiveness.mp. | 95585 |
| 36 | M?ID.mp. | 112825 |
| 37 | (clinical* adj2 (meaningful or important*)).mp. | 71624 |
| 38 | (minim* adj2 important*).mp. | 4620 |
| 39 | exp psychometry/ or psychometr*.mp. | 82229 |
| 40 | Sensitiv*.mp. | 1476430 |
| 41 | or/32-40 | 2700363 |
| 42 | 4 and 26 and 41 | 1951 |
| 43 | 31 or 42 | 2596 |

Table S3: Search strategy for Ovid MEDLINE(R) In-Process & Other Non-Indexed Citations and Ovid MEDLINE(R) 1946 to Present, Accessed 15-September-2015

| # | Searches | Results |
| --- | --- | --- |
| 1 | exp Infertility, Female/ | 24734 |
| 2 | Infertility/ or infertil*.mp. | 73900 |
| 3 | fertil*.mp. | 166418 |
| 4 | or/1-3 | 210516 |
| 5 | (EQ-5D or EQ5D or Euroqol).mp. | 5209 |
| 6 | (Health utilities index or HUI).mp. | 1180 |
| 7 | (time trade off or ("TTO" adj2 "time trade")).mp. | 870 |
| 8 | standard gamble.mp. | 737 |
| 9 | (15D or 16D or 17D).mp. | 2184 |
| 10 | (medical outcomes survey or MOS).mp. | 5893 |
| 11 | (Quality of wellbeing index or QWB).mp. | 188 |
| 12 | FertiQoL.mp. | 20 |
| 13 | (Fertility problem Inventory or FpI).mp. | 996 |
| 14 | (Hopkins Symptom checklist or SCL).mp. | 5931 |
| 15 | WHOQOL.mp. | 1652 |
| 16 | Enrich Inventory.mp. | 4 |
| 17 | Sickness impact profile.mp. | 7187 |
| 18 | (Sexual Functioning Questionnaire or SFQ).mp. | 181 |
| 19 | ("quality of well-being scale" or "quality of wellbeing scale").mp. | 166 |
| 20 | Questionnaires/ or questionnaire*.mp. | 511600 |
| 21 | Short form.mp. | 20468 |
| 22 | Instrument*.mp. | 229696 |
| 23 | (Patient reported outcome* or PRO).mp. | 145255 |
| 24 | PROM.mp. | 1601 |
| 25 | scale*.mp. | 577142 |
| 26 | or/5-25 | 1334701 |
| 27 | (QoL or HRQoL or HRQL).mp. | 33879 |
| 28 | exp "Quality of Life"/ or quality of life.mp | 223731 |
| 29 | Treatment satisfaction.mp. | 1839 |
| 30 | or/ 27-29 | 225726 |
| 31 | 4 and 26 and 30 | 415 |
| 32 | exp "Reproducibility of Results"/ or reproducib*.mp. | 392290 |
| 33 | reliab*.mp. | 343896 |
| 34 | valid*.mp. | 493083 |
| 35 | responsiveness.mp. | 85849 |
| 36 | M?ID.mp. | 89965 |
| 37 | (clinical* adj2 (meaningful or importan*)).mp. | 55260 |
| 38 | (minim* adj2 importan*).mp. | 3271 |
| 39 | Psychometrics/ or psychometr*.mp. | 72018 |
| 40 | Sensitiv*.mp. | 1317741 |
| 41 | or/32-40 | 2326260 |
| 42 | 4 and 26 and 41 | 1277 |
| 43 | 31 or 42 | 1605 |

Table S4: Search strategy for Cochrane Central Register of Controlled Trials August 2015, Cochrane Database of Systematic Reviews 2005 to August 2015, Database of Abstracts of Reviews of Effects 2nd Quarter 2015, Health Technology Assessment 3rd Quarter 2015, and NHS Economic Evaluation Database 2^nd^ Quarter 2015, Accessed 15-September-2015

| # | Searches | Results |
| --- | --- | --- |
| 1 | exp Infertility, Female/ | 977 |
| 2 | Infertility/ or infertil*.mp. | 3972 |
| 3 | fertil*.mp. | 5139 |
| 4 | or/1-3 | 7044 |
| 5 | (EQ-5D or EQ5D or Euroqol).mp. | 2588 |
| 6 | (Health utilities index or HUI).mp. | 339 |
| 7 | (time trade off or ("TTO" adj2 "time trade")).mp. | 452 |
| 8 | standard gamble.mp. | 273 |
| 9 | (15D or 16D or 17D).mp. | 133 |
| 10 | (medical outcomes survey or MOS).mp. | 822 |
| 11 | (Quality of wellbeing index or QWB).mp. | 56 |
| 12 | FertiQoL.mp. | 2 |
| 13 | (Fertility problem Inventory or FpI).mp. | 124 |
| 14 | (Hopkins Symptom checklist or SCL).mp. | 785 |
| 15 | WHOQOL.mp. | 232 |
| 16 | Enrich Inventory.mp. | 0 |
| 17 | Sickness impact profile.mp. | 802 |
| 18 | (Sexual Functioning Questionnaire or SFQ).mp. | 47 |
| 19 | ("quality of well-being scale" or "quality of wellbeing scale").mp. | 85 |
| 20 | Questionnaires/ or questionnaire*.mp. | 47943 |
| 21 | Short form.mp. | 5057 |
| 22 | Instrument*.mp. | 14894 |
| 23 | (Patient reported outcome* or PRO).mp. | 5433 |
| 24 | PROM.mp. | 253 |
| 25 | scale*.mp. | 82536 |
| 26 | or/5-25 | 129895 |
| 27 | (QoL or HRQoL or HRQL).mp. | 7891 |
| 28 | exp "Quality of Life"/ or quality of life.mp | 45447 |
| 29 | Treatment satisfaction.mp. | 837 |
| 30 | or/ 27-29 | 46369 |
| 31 | 4 and 26 and 30 | 192 |
| 32 | exp "Reproducibility of Results"/ or reproducib*.mp. | 13378 |
| 33 | reliab*.mp. | 33173 |
| 34 | valid*.mp. | 45468 |
| 35 | responsiveness.mp. | 4574 |
| 36 | M?ID.mp. | 4893 |
| 37 | (clinical* adj2 (meaningful or importan*)).mp. | 9998 |
| 38 | (minim* adj2 importan*).mp. | 1023 |
| 39 | Psychometrics/ or psychometr*.mp. | 4659 |
| 40 | Sensitiv*.mp. | 60807 |
| 41 | or/32-40 | 113621 |
| 42 | 4 and 26 and 41 | 402 |
| 43 | 31 or 42 | 430 |
