# Supplementary material for: A review of patient-reported outcome measures to assess female infertility-related quality of life
Source: Health Qual Life Outcomes. 2017 Apr 27;15:86. doi: 10.1186/s12955-017-0666-0 (PMC5408488; doi:10.1186/s12955-017-0666-0)
Supplement: Supplementary file 3 — Detailed review of PRO measures. Three tables presenting further methodological details of the PRO review. (DOCX 53 kb) [file 12955_2017_666_MOESM3_ESM.docx]

### Detailed review of PRO measures: Further methods

Table S5: Content validity review checklist

| Category | Example questions |
| --- | --- |
| Instrument development | - Documented evidence that patients representative of the intended population of use were involved in item generation - Documented evidence that patients representative of the intended population of use were involved in content validity testing (e.g. cognitive debriefing interviews) - Documentation of previous versions of the instrument and rationale for item reduction. |
| Conceptual framework | - Documentation of a conceptual framework - Documented evidence that each item measures a single concept |
| Instructions | - Documented evidence for patient understanding and consistent interpretation of diary instructions - Availability of specific instructions relevant to the electronic mode of the instrument |
| Response scale and options | - Documented evidence for patient understanding and consistent interpretation of response scale and response options - Documented evidence that the response options fit the target concepts - Documented evidence that the response options are evenly distributed - Evidence of use of a scale appropriate to the response options |
| Recall period | - Documented evidence that the recall period is appropriate for measurement concepts - Documented evidence that the recall period is appropriate for intended context of use and population of use |
| Item wording | - Documented evidence for patient understanding and consistent interpretation of items |
| Format and layout | - Documented evidence that the administration mode is suitable for the target population |
| Scoring | - Evidence for conceptually relevant scoring at the item level and domain level - Availability of a user manual or scoring guide for consistency in data analyses |

Abbreviations: FDA, Food and Drug Administration; PRO, patient reported outcome

Table S6: Psychometric review checklist

| Category | Property | Definitions |
| --- | --- | --- |
| Reliability ([22](#_ENREF_22)) | Internal (Cronbach’s α) | Extent to which items within a domain measure the same concept |
|  | External (test-retest) | Stability of scores over time when no change is expected in the concept of interest |
| Criterion validity ([22](#_ENREF_22)) | Concurrent | Strength of correlation with scores of a ‘gold standard’ measure |
|  | Predictive | Accuracy of prediction of scores of a ‘gold standard’ measure |
| Construct validity ([22](#_ENREF_22)) | Convergent/ divergent | Evidence that relationships among items, domains, and concepts conform to a priori hypotheses concerning logical relationships that should exist with measures of related concepts |
|  | Structural | Structure of the instrument determined as hypothesised |
|  | Known-groups | Degree to which the PRO instrument can distinguish among groups hypothesized *a priori* to be different |
| Ability to detect change ([22](#_ENREF_22)) | Responsiveness/ sensitivity | Evidence that a PRO instrument can identify differences in scores over time in individuals or groups who have changed with respect to the measurement concept |
|  | Clinically important differences (CID) | Group-level change: the difference in scores between 2 treatment groups that can be considered clinically relevant  Individual level change: the amount of change a patient would have to report to indicate that a relevant treatment benefit has bene experienced |

Table S7: Checklist of practical considerations

| Category | Example questions |
| --- | --- |
| Cross-cultural feasibility | - Is the instrument available in different languages and cultural adaptations? - Are these translations validated appropriately? |
| Patient burden | - Are there any implications for patient burden due to the length of the instrument? |
| Format | - Does the instrument need to be completed using pen and paper, or is there an electronic version available? |
