# Supplementary material for: A review of patient-reported outcome measures to assess female infertility-related quality of life
Source: Health Qual Life Outcomes. 2017 Apr 27;15:86. doi: 10.1186/s12955-017-0666-0 (PMC5408488; doi:10.1186/s12955-017-0666-0)
Supplement: Supplementary file 4 — Publications excluded at second pass (screened by full paper). Table listing each publication excluded during second pass of the literature review, detailing first author, title, citation, and the reason for exclusion. (DOCX 82 kb) [file 12955_2017_666_MOESM4_ESM.docx]

### Publications excluded at second pass (screened by full paper)

Table S8: List of publications excluded at second pass (screened by full paper), n=213

| First author et al | Title | Citation | Reason for exclusion |
| --- | --- | --- | --- |
| Aarts, J. W. M. et al, 2010 | Quality of life measure as an extra tool for delivering patient centred care | Human Reproduction. 2010; 25():i252-i253 | Setting not treatment-specific |
| Aarts, J. W. M. et al, 2012 | How patient-centred care relates to patients quality of life and distress: A study in 427 women experiencing infertility | Human Reproduction. 2012; 27(2):488-495 | Setting not treatment-specific |
| Abedi, G. et al, 2014 | The relationship between quality of life and marriage satisfaction in infertile couples using path analysis. [Persian] | Journal of Mazandaran University of Medical Sciences. 2014; 24(117):184-193 | Setting not treatment-specific |
| Ajdukovic, D. et al, 2013 | The interaction of personal and partner’s correlates of fertility-related quality of life | Human Reproduction. 2013; 28():i271-i272 | Setting not treatment-specific |
| Akyuz, A. et al, 2009 | Development and validation of an infertility distress scale for Turkish women | Molecular Human Reproduction. 2009; 24(Suppl1):i224-i225 | Study purpose: PRO development methodology paper, no results reported |
| Algul, O. et al, 2014 | Investigation of sexual activity dysfunction and quality of life of the couples having infertility problem. [Turkish] | Turkiye Klinikleri Jinekoloji Obstetrik. 2014; 24(3):171-178 | Setting not treatment-specific |
| Algul, O. et al, 2014 | Investigation of sexual activity dysfunction and quality of life of the couples having infertility problem | European Journal of Contraception and Reproductive Health Care. 2014; 19():S222-S223 | Setting not treatment-specific |
| Alijanpoor, M. et al, 2014 | The relationship between spiritual health and quality of life in infertile women | International Journal of Fertility and Sterility. 2014; 8():103 | Setting not treatment-specific |
| Alimanesh, N. et al, 2013 | Self-efficacy of infertile women and related factors | Iranian Journal of Reproductive Medicine. 2013; 11():33 | Setting not treatment-specific |
| Aliyeh, G. et al, 2007 | Quality of life and its correlates among a group of infertile Iranian women | Medical science monitor: international medical journal of experimental and clinical research. 2007; 13(7):CR313-317 | Setting not treatment-specific |
| Alvarez, S. et al, 2012 | First French national survey on lifestyle and toxic factors in infertile couples. [French] | Gynecologie Obstetrique Fertilite. 2012; 40(12):765-771 | Setting not treatment-specific |
| Amanati, L. et al, 2010 | Quality of life and influencing factors among infertile women. [Arabic] | Iranian Journal of Obstetrics, Gynaecology and Infertility. 2010; 12(4):25-31 | Setting not treatment-specific |
| Andrews, F. M. et al, 1991 | Stress from infertility, marriage factors, and subjective well-being of wives and husbands | Journal of health and social behaviour. 1991; 32(3):238-253 | Methodology: Does not use a PRO |
| Andrews, F. M. et al, 1992 | Is fertility-problem stress different? The dynamics of stress in fertile and infertile couples | Fertility and Sterility. 1992; 57(6):1247-1253 | Study purpose: PRO development methodology paper, no results reported |
| Arab, K. et al, 2012 | Admissibility investigation and validation of infertility distress scale (IDS) in Iranian infertile women | International Journal of Fertility and Sterility. 2012; 6(1):37-44 | Study purpose: PRO development methodology paper, no results reported |
| Ashraf, D. M. et al, 2014 | Effect of infertility on the quality of life, a cross- sectional study | Journal of Clinical and Diagnostic Research JCDR. 2014; 8(10):OC13-5 | Setting not treatment-specific |
| Baczkowski, T. et al, 2007 | Sex life among infertile couples treated with artificial reproductive techniques. [Polish] | Family Medicine and Primary Care Review. 2007; 9(3):375-377 | Study purpose: Not related to QoL/treatment satisfaction |
| Bakhshayesh, A. R. et al, 2012 | A comparison of general health and coping strategies in fertile and infertile women in Yazd | Iranian Journal of Reproductive Medicine. 2012; 10(6):601-6 | Setting not treatment-specific |
| Bhaskar, S. et al, 2012 | The validation of quality of life scale for men with Involuntary Childlessness (TLMK) - Tamil version | Indian Journal of Psychiatry. 2012; 54():S75 | Abstract only; population not fully described |
| Bhaskar, S. et al, 2014 | Life experiences and quality of life of involuntarily childless men in treatment and adoptive fathers | Journal of Reproductive and Infant Psychology. 2014; 32(5):497-507 | Setting not treatment-specific |
| Bidzan, M. et al, 2011 | [Personality traits and the feeling of loneliness of women treated for infertility] | Ginekologia Polska. 2011; 82(7):508-13 | Study purpose: Not related to QoL/treatment satisfaction |
| Biringer, E. et al, 2010 | Common mental disorders and fertility problems in the normal population | European Psychiatry. 2010; 25(): | Setting not treatment-specific |
| Boivin, J. et al, 2011 | The fertility quality of life (FertiQoL) tool: Development and general psychometric properties | Human Reproduction. 2011; 26(8):2084-2091 | Study purpose: PRO development methodology paper, no results reported |
| Bolsoy, N. et al, 2010 | Differences in quality of life between infertile women and men in Turkey | JOGNN - Journal of Obstetric, Gynaecologic, and Neonatal Nursing. 2010; 39(2):191-198 | Setting not treatment-specific |
| Bresnick, E. et al, 1979 | The role of counselling in infertility | Fertility & Sterility. 1979; 32(2):154-6 | Setting not treatment-specific |
| Brod, M. et al, 2009 | Improving clinical understanding of the effect of ovarian stimulation on women's lives | Reproductive Biomedicine Online. 2009; 18(3):391-400 | Study purpose: PRO development methodology paper, no results reported |
| Bromham, D. R. et al, 1989 | Psychometric evaluation of infertile couples. (Preliminary Findings) | Journal of Reproductive and Infant Psychology. 1989; 7(4):195-202 | Setting not treatment-specific |
| Burnham, K. E. et al, 2014 | Social concern among women reporting trauma symptoms related to infertility | Psychosomatic Medicine. 2014; 76(3):A-78 | Setting not treatment-specific |
| Carter, J. et al, 2010 | A cross-sectional study of the psychosexual impact of cancer-related infertility in women: Third-party reproductive assistance | Journal of Cancer Survivorship. 2010; 4(3):236-246 | Setting not treatment-specific |
| Carter, A. et al, 2011 | Female infertility patients and their male partners under-report anxiety and depression | Fertility and Sterility. 2011; 1)():S24 | Setting not treatment-specific |
| Carter, J. et al, 2011 | A cross-sectional cohort study of infertile women awaiting oocyte donation: The emotional, sexual, and quality-of-life impact | Fertility and Sterility. 2011; 95(2):711-716.e1 | Setting not treatment-specific |
| Chachamovich, J. et al, 2009 | Congruence of quality of life among infertile men and women: Findings from a couple-based study | Human Reproduction. 2009; 24(9):2151-2157 | Setting not treatment-specific |
| Chachamovich, J. et al, 2010 | Agreement on perceptions of quality of life in couples dealing with infertility | JOGNN - Journal of Obstetric, Gynaecologic, and Neonatal Nursing. 2010; 39(5):557-565 | Setting not treatment-specific |
| Chachamovich, J. et al, 2010 | Psychological distress as predictor of quality of life in men experiencing infertility: a cross-sectional survey | Reproductive Health. 2010; 7():3 | Setting not treatment-specific |
| Chi, H. J. et al, 2014 | Level of psychological distress in Korean infertile women, and correlation between psychological distress and quality of life; The first validation study of Korean Ferti-QoL | Fertility and Sterility. 2014; 1():e247 | Setting not treatment-specific |
| Choobforoushzade, A. et al, 2011 | The effectiveness of cognitive behavioural stress management therapy on quality of life in infertile women | Iranian Journal of Obstetrics, Gynecology and Infertility. 2011; 14(1):14-21 | Setting not treatment-specific |
| Clua, E. et al, 2011 | Cross-border reproductive care and psychological distress | Human Reproduction. 2011; 26():i148-i149 | Setting not treatment-specific |
| Coughlan, C. et al, 2014 | A comparison of psychological stress among women with and without reproductive failure | International Journal of Gynecology and Obstetrics. 2014; 124(2):143-147 | Setting not treatment-specific |
| Cousineau, T. M. et al, 2006 | Development and validation of the Infertility Self-Efficacy scale | Fertility and Sterility. 2006; 85(6):1684-1696 | Study purpose: PRO development methodology paper, no results reported |
| Coyle, M. et al, 2005 | A survey comparing TCM diagnosis, health status and medical diagnosis in women undergoing assisted reproduction | Acupuncture in Medicine. 2005; 23(2):62-69 | Setting not treatment-specific |
| Cristiana, H. et al, 2010 | Gender differences in quality of life of infertile couples and in the intensity of dysfunctional attitudes | Journal of Psychosomatic Obstetrics and Gynecology. 2010; 31():81 | Study purpose: Postnatal QoL |
| Cserepes, R. E. et al, 2013 | Connections between fertiqol and fertility problem inventory (FPI) in a Hungarian sample of couples facing involuntary childlessness | Human Reproduction. 2013; 28():i273 | Setting not treatment-specific |
| Cserepes, R. E. et al, 2013 | Effects of gender roles, child wish motives, subjective well-being, and marital adjustment on infertility-related stress: A preliminary study with a Hungarian sample of involuntary childless men and women | Archives of Gynecology and Obstetrics. 2013; 288(4):925-932 | Setting not treatment-specific |
| Cserepes, R. E. et al, 2014 | Infertility specific quality of life and gender role attitudes in German and Hungarian involuntary childless couples | Geburtshilfe und Frauenheilkunde. 2014; 74(11):1009-1015 | Setting not treatment-specific |
| Cserepes, R. E. et al, 2014 | Characteristics of infertility specific quality of life in Hungarian couples. [Hungarian] | Orvosi Hetilap. 2014; 155(20):783-788 | Setting not treatment-specific |
| Cserepes, R. E. et al, 2015 | [Impact of depressive symptomatology in Hungarian infertile couples] | Psychiatria Hungarica. 2015; 30(1):50-9 | Setting not treatment-specific |
| De Bot, C. M. A. et al, 2005 | QUOTE fertility: Quality of care according to clients who have undergone fertility treatment. [Dutch] | Nederlands Tijdschrift voor Obstetrie en Gynaecologie. 2005; 118(2):33-37 | Study purpose: Not related to QoL/treatment satisfaction |
| Dilbaz, B. et al, 2012 | Health related quality of life among different PCOS phenotypes of infertile women | Journal of the Turkishgerman Gynecological Association. 2012; 13(4):247-52 | Setting not treatment-specific |
| Domar, A. D. et al, 1992 | Psychological improvement in infertile women after behavioral treatment: A replication | Fertility and Sterility. 1992; 58(1):144-147 | Setting not treatment-specific |
| Domar, A. D. et al, 1993 | The psychological impact of infertility: A comparison with patients with other medical conditions | Journal of Psychosomatic Obstetrics and Gynaecology. 1993; 14(SUPPL.):45-52 | Setting not treatment-specific |
| Domar, A. D. et al, 2015 | Exploratory randomized trial on the effect of a brief psychological intervention on emotions, quality of life, discontinuation, and pregnancy rates in in vitro fertilization patients | Fertility and Sterility. 2015; 104(2):440-451.e7 | Setting not treatment-specific |
| Donarelli, Z. et al, 2015 | Assessing infertility-related stress: The factor structure of the Fertility Problem Inventory in Italian couples undergoing infertility treatment | Journal of Psychosomatic Obstetrics and Gynecology. 2015; 36(2):58-65 | Study purpose: PRO development methodology paper, no results reported |
| Dong, Y. Z. et al, 2013 | Correlative analysis of social support with anxiety and depression in men undergoing in vitro fertilization embryo transfer for the first time | Journal of International Medical Research. 2013; 41(4):1258-1265 | Setting not treatment-specific |
| Drosdzol, A. et al, 2008 | Quality of life and sexual functioning of Polish infertile couples | European Journal of Contraception and Reproductive Health Care. 2008; 13(3):271-281 | Setting not treatment-specific |
| Ebbesen, S. M. et al, 2009 | Stressful life events are associated with a poor in-vitro fertilization (IVF) outcome: a prospective study | Human Reproduction. 2009; 24(9):2173-82 | Setting not treatment-specific |
| El Kissi, Y. et al, 2014 | Quality of life of infertile Tunisian couples and differences according to gender | International Journal of Gynaecology & Obstetrics. 2014; 125(2):134-7 | Setting not treatment-specific |
| El Kissi, Y. et al, 2013 | General psychopathology, anxiety, depression and self-esteem in couples undergoing infertility treatment: A comparative study between men and women | European Journal of Obstetrics Gynecology and Reproductive Biology. 2013; 167(2):185-189 | Setting not treatment-specific |
| El-Messidi, A. et al, 2004 | Effects of repeated treatment failure on the quality of life of couples with infertility | Journal of obstetrics and gynaecology Canada: JOGC = Journal d'obstetrique et gynecologie du Canada: JOGC. 2004; 26(4):333-336 | Full paper unavailable due to copyright restrictions; no useable data presented in abstract |
| Erfanian Ahmadpoor, M. et al, 2012 | A comparative Study of the quality of life between man and woman in infertile couples | Iranian Journal of Reproductive Medicine. 2012; 10():114-115 | Setting not treatment-specific |
| Erfanian Ahmadpoor, M. et al, 2013 | A comparative Study of the quality of life between man and woman in infertile couples | Iranian Journal of Reproductive Medicine. 2013; 11():46-47 | Setting not treatment-specific |
| Fardiazar, Z. et al, 2012 | Irrational parenthood cognitions and health-related quality of life among infertile women | International Journal of General Medicine. 2012; 5():591-596 | Setting not treatment-specific |
| Fassino, S. et al, 2002 | Anxiety, depression and anger suppression in infertile couples: A controlled study | Human Reproduction. 2002; 17(11):2986-2994 | Setting not treatment-specific |
| Fatoye, F. O. et al, 2009 | Psychological profile of spouses of women with infertility in Nigeria | African journal of medicine and medical sciences. 2009; 38(1):63-69 | Setting not treatment-specific |
| Fazel, A. et al, 2014 | Fertility outcome and quality of life at long term follow up of combined embolization and selective minimally invasive myomectomy after mri (CESAM) | Gynecological Surgery. 2014; 1():70-72 | Study purpose: Postnatal QoL |
| Fekkes, M. et al, 2003 | Health-related quality of life in relation to gender and age in couples planning IVF treatment | Human Reproduction. 2003; 18(7):1536-43 | Setting not treatment-specific |
| Franco, J. G. et al, 2001 | Psychometry of stress in infertile couples. [Portuguese] | Jornal Brasileiro de Reproducao Assistida. 2001; 5(2):55-59 | Setting not treatment-specific |
| Franco, J. G. et al, 2002 | Psychological evaluation test for infertile couples | Journal of Assisted Reproduction & Genetics. 2002; 19(6):269-73 | Setting not treatment-specific |
| Franco, J. G. et al, 2004 | Comparison of the psychological evaluation test and classical psychoanalysis in infertile women | Reproductive BioMedicine Online. 2004; 8(1):8-13 | Setting not treatment-specific |
| Franco, J. G. et al, 2004 | Comparison of the psychological evaluation test and classical psychoanalysis in infertile women. [Portuguese] Comparacao, em mulheres inferteis, de teste de avaliacao psicologica com a analise psicanalitica classica | Reprod Biomed Online. 2004 Jan;8(1):8-13. | Setting not treatment-specific |
| Fu, B. et al, 2015 | Development and validation of an Infertility Stigma Scale for Chinese women | Journal of Psychosomatic Research. 2015; 79(1):69-75 | Study purpose: Not related to QoL/treatment satisfaction |
| Galhardo, A. et al, 2013 | The mediator role of emotion regulation processes on infertility-related stress | Journal of Clinical Psychology in Medical Settings. 2013; 20(4):497-507 | Setting not treatment-specific |
| Galhardo, A. et al, 2013 | Dealing with the challenges of infertility: The Portuguese version of the infertility self-efficacy scale | Atencion Primaria. 2013; 45():143-144 | Study purpose: PRO development methodology paper, no results reported |
| Galhardo, A. et al, 2013 | Measuring self-efficacy to deal with infertility: Psychometric properties and confirmatory factor analysis of the Portuguese version of the infertility self-efficacy scale | Research in Nursing and Health. 2013; 36(1):65-74 | Study purpose: PRO development methodology paper, no results reported |
| Gameiro, S. et al, 2013 | Patient centred care in infertility health care: Direct and indirect associations with wellbeing during treatment | Patient Education and Counselling. 2013; 93(3):646-654 | Setting not treatment-specific |
| Glover, L. et al, 1999 | Development of the fertility adjustment scale | Fertility and Sterility. 1999; 72(4):623-628 | Study purpose: PRO development methodology paper, no results reported |
| Goldschmidt, S. et al, 2001 | Life satisfaction of infertile couples during in vitro fertilization in relation to the treatment outcome. [German] | Zeitschrift fur Klinische Psychologie, Psychiatrie und Psychotherapie. 2001; 49(2):197-220 | Study purpose: Postnatal QoL |
| Goldschmidt, S. et al, 2003 | Relationship between satisfaction with life and treatment outcome after IVF in involuntarily childless couples. [German] | Reproduktionsmedizin. 2003; 19(1):30-39 | Study purpose: Postnatal QoL |
| Gourounti, K. et al, 2011 | Psychometric properties and factor structure of the Fertility Problem Inventory in a sample of infertile women undergoing fertility treatment | Midwifery. 2011; 27(5):660-667 | Study purpose: PRO development methodology paper, no results reported |
| Gourounti, K. et al, 2012 | Perception of control, coping and psychological stress of infertile women undergoing IVF | Human Reproduction. 2012; 27(): | Setting not treatment-specific |
| Gourounti, K. et al, 2012 | Appraisal of Life Events scale in a sample of Greek infertile women undergoing fertility treatment: A confirmatory factor analysis | Midwifery. 2012; 28(4):385-390 | Study purpose: PRO development methodology paper, no results reported |
| Greil, A. L. et al, 2011 | Variation in distress among women with infertility: Evidence from a population-based sample | Human Reproduction. 2011; 26(8):2101-2112 | Setting not treatment-specific |
| Gullo, S. et al, 2013 | The fertility problem inventory (FPI): A validation study of the Italian version | Human Reproduction. 2013; 28():i264-i265 | Study purpose: PRO development methodology paper, no results reported |
| Haica, C., 2012 | Assessing relative importance of FertiQoL dimensions on General Quality of Life, DAS, PDE, USAQ | Human Reproduction. 2012; 27(Suppl2):ii268-ii273 | Study purpose: PRO development methodology paper, no results reported |
| Hammar, O. et al, 2013 | Autoantibodies and gastrointestinal symptoms in infertile women in relation to in vitro fertilization | BMC Pregnancy and Childbirth. 2013; 13(1):201 | Study purpose: Not related to QoL/treatment satisfaction |
| Harf-Kashdaei, E. et al, 2007 | Antenatal moods regarding self, baby, and spouse among women who conceived by in vitro fertilization | Fertility and Sterility. 2007; 87(6):1306-1313 | Study purpose: Postnatal QoL |
| Hassanin, I. M. et al, 2010 | Primary infertility and health-related quality of life in Upper Egypt | International Journal of Gynaecology & Obstetrics. 2010; 110(2):118-21 | Setting not treatment-specific |
| Heredia, M. et al, 2013 | Quality of life and predictive factors in patients undergoing assisted reproduction techniques | European Journal of Obstetrics Gynecology and Reproductive Biology. 2013; 167(2):176-180 | Setting not treatment-specific |
| Herrmann, D. et al, 2011 | Resilience in infertile couples acts as a protective factor against infertility-specific distress and impaired quality of life | Journal of Assisted Reproduction and Genetics. 2011; 28(11):1111-1117 | Setting not treatment-specific |
| Hoffman, J. R. et al, 2013 | Fertility knowledge is lower among women from socio-culturally diverse, lower-income communities in the United States | Fertility and Sterility. 2013; 1():S408 | Study purpose: Not related to QoL/treatment satisfaction |
| Holter, H. et al, 2014 | Patient-centred quality of care in an IVF programme evaluated by men and women | Human Reproduction. 2014; 29(12):2695-2703 | Study purpose: Not related to QoL/treatment satisfaction |
| Holter, H. et al, 2014 | Quality of care in an IVF programme from a patient's perspective: Development of a validated instrument | Human Reproduction. 2014; 29(3):534-547 | Study purpose: Not related to QoL/treatment satisfaction |
| Hsu, Y. L. et al, 2002 | Evaluations of emotional reactions and coping behaviours as well as correlated factors for infertile couples receiving assisted reproductive technologies | The journal of nursing research : JNR. 2002; 10(4):291-302 | Setting not treatment-specific |
| Hsu, P. Y. et al, 2013 | The fertility quality of life (FertiQoL) questionnaire in Taiwanese infertile couples | Taiwanese Journal of Obstetrics and Gynecology. 2013; 52(2):204-209 | Setting not treatment-specific |
| Huppelschoten, A. G. et al, 2011 | Effects of a multifaceted approach on improvement of patient centredness in fertility care, a study protocol | Human Reproduction. 2011; 26():i270 | Study purpose: Not related to QoL/treatment satisfaction |
| Huppelschoten, A. G. et al, 2012 | Improving patient-centeredness of fertility care using a multifaceted approach: study protocol for a randomized controlled trial | Trials. 2012; 13(1):175 | Protocol only: No study data |
| Huppelschoten, A. G. et al, 2013 | Differences in quality of life and emotional status between infertile women and their partners | Human Reproduction. 2013; 28(8):2168-76 | Setting not treatment-specific |
| Huppelschoten, A. G. et al, 2013 | Do infertile women and their partners have equal experiences with fertility care? | Fertility & Sterility. 2013; 99(3):832-8 | Study purpose: Not related to QoL/treatment satisfaction |
| Huppelschoten, A. G. et al, 2013 | The relation between drop out and patient-centredness in fertility care | Human reproduction (Oxford, England). 2013; 28(Suppl1):i33-i34 | Study purpose: Not related to QoL/treatment satisfaction |
| Izhar, R. et al, 2012 | Comparison of saline hysterosonography & hysterosalpingogram in assessment of fallopian tube patency & intrauterine abnormalities in infertility assessment | International Journal of Gynecology and Obstetrics. 2012; 119():S377-S378 | Study purpose: Not related to QoL/treatment satisfaction |
| Jafari, H. et al, 2013 | The association of self-efficacy with health locus of control and psychological distress in infertile women | International Journal of Fertility and Sterility. 2013; 7():41 | Setting not treatment-specific |
| James, B. et al, 1982 | Psychological well-being as an outcome variable in the treatment of infertility by Clomiphine | British Journal of Medical Psychology. 1982; 55(Pt 4):375-7 | Study purpose: Postnatal QoL |
| Johansson, M. et al, 2009 | Quality of life for couples 4-5.5 years after unsuccessful IVF treatment | Acta Obstetricia et Gynecologica Scandinavica. 2009; 88(3):291-300 | Study purpose: Postnatal QoL |
| Johansson, M. et al, 2010 | Gender perspective on quality of life, comparisons between groups 4-5.5 years after unsuccessful or successful IVF treatment | Acta Obstetricia et Gynecologica Scandinavica. 2010; 89(5):683-91 | Study purpose: Postnatal QoL |
| Kadota, T. et al, 2012 | Personality traits associated with the psychological burden on infertile women: Tolerance of ambiguity, cognitive control, resilience | Human Reproduction. 2012; 27(): | Study purpose: Not related to QoL/treatment satisfaction |
| Kakatsaki, D. et al, 2009 | Alexithymia is positively associated with the outcome of in vitro fertilization (IVF) treatment | Psychological reports. 2009; 105(2):522-532 | Setting not treatment-specific |
| Kalhor, M. et al, 2014 | Quality of life and related factors among infertile women | Iranian Journal of Reproductive Medicine. 2014; 1():129 | Setting not treatment-specific |
| Kamalifard, M. et al, 2012 | Health-related Quality of life and its predictive factors in infertile women | Iranian Journal of Reproductive Medicine. 2012; 10():34-35 | Setting not treatment-specific |
| Karabulut, A. et al, 2013 | Predictors of fertility quality of life (FertiQoL) in infertile women: Analysis of confounding factors | European Journal of Obstetrics Gynecology and Reproductive Biology. 2013; 170(1):193-197 | Setting not treatment-specific |
| Keramat, A. et al, 2014 | Quality of life and its related factors in infertile couples | Journal of Research in Health Sciences. 2014; 14(1):57-63 | Setting not treatment-specific |
| Khayata, G. M. et al, 2003 | Factors influencing the quality of life of infertile women in United Arab Emirates | International Journal of Gynecology and Obstetrics. 2003; 80(2):183-188 | Setting not treatment-specific |
| Khorram, S. et al, 2013 | Mediation role of negative emotions on the relationship between marital conflict and physical health in infertile women | Iranian Journal of Reproductive Medicine. 2013; 11():113 | Setting not treatment-specific |
| Khorram, S. et al, 2013 | The causal model of the marital conflict, negative emotions, and sexual function in infertile women | Iranian Journal of Reproductive Medicine. 2013; 11():112 | Setting not treatment-specific |
| Khorram, S. et al, 2013 | An investigation of the causal model of the relations between the marital conflict, physical health and sexual function in infertile women | Iranian Journal of Reproductive Medicine. 2013; 11():112 | Setting not treatment-specific |
| Khorram, S. et al, 2013 | The relationship between marital conflict, negative emotion, physical health and sexual function in infertile women | Iranian Journal of Reproductive Medicine. 2013; 11():111-112 | Setting not treatment-specific |
| Kim, J. H. et al, 2013 | A structural model for quality of life of infertile women | Journal of Korean Academy of Nursing. 2013; 43(3):312-320 | Setting not treatment-specific |
| Kim, J. H. et al, 2014 | Validation of a Korean version of fertility problem inventory | Asian Nursing Research. 2014; 8(3):207-212 | Study purpose: PRO development methodology paper, no results reported |
| Klemetti, R. et al, 2010 | Infertility, mental disorders and well-being - A nationwide survey | Acta Obstetricia et Gynecologica Scandinavica. 2010; 89(5):677-682 | Study purpose: Postnatal QoL |
| Klonoff-Cohen, H. et al, 2007 | Validation of a new scale for measuring concerns of women undergoing assisted reproductive technologies (CART) | Journal of Health Psychology. 2007; 12(2):352-356 | Study purpose: PRO development methodology paper, no results reported |
| Kolesnikov, D. B. et al, 2013 | [The psychic state of women suffering infertility in the old reproductive age]. [Russian] | Klinicheskaia meditsina. 2013; 91(6):38-41 | Setting not treatment-specific |
| Koropatnic, S. et al, 1993 | Infertility: A non-event transition | Fertility and Sterility. 1993; 59(1):163-171 | Setting not treatment-specific |
| Kudesia, R. et al, 2014 | Quality of life and psychosocial impact of infertility in Uganda | Reproductive Sciences. 2014; 1():87A | Setting not treatment-specific |
| Kuivasaari-Pirinen, P. et al, 2014 | Outcome of assisted reproductive technology (ART) and subsequent self-reported life satisfaction | PloS one. 2014; 9(11):e112540 | Study purpose: Postnatal QoL |
| Lasheras, G. et al, 2013 | Cross-border reproductive care and psychological distress | Archives of Women's Mental Health. 2013; 16():S68 | Setting not treatment-specific |
| Lau, J. T. et al, 2008 | Infertility-related perceptions and responses and their associations with quality of life among rural chinese infertile couples | Journal of Sex & Marital Therapy. 2008; 34(3):248-67 | Setting not treatment-specific |
| Ledger, W. et al, 2013 | Impact of digital home ovulation test usage on stress, psychological wellbeing and quality of life during evaluation of subfertility: A randomised controlled trial | Fertility and Sterility. 2013; 1():S411 | Setting not treatment-specific |
| Lee, T. Y. et al, 2000 | Psychosocial response of Chinese infertile husbands and wives | Archives of Andrology. 2000; 45(3):143-8 | Setting not treatment-specific |
| Lee, T. Y. et al, 2000 | Development of the coping scale for infertile couples | Archives of Andrology. 2000; 45(3):149-154 | Study purpose: Not related to QoL/treatment satisfaction |
| Letourneau, J. et al, 2013 | Novel infertility screening questionnaire predicts need for mental health counselling in patients presenting for infertility evaluation | Fertility and Sterility. 2013; 1():S415 | Setting not treatment-specific |
| Lewis, A. M. et al, 2013 | Less depressed or less forthcoming? Self-report of depression symptoms in women preparing for in vitro fertilization | Archives of Women's Mental Health. 2013; 16(2):87-92 | Setting not treatment-specific |
| Lopes, V. et al, 2014 | Are patients at risk for psychological maladjustment during fertility treatment less willing to comply with treatment? Results from the Portuguese validation of the SCREENIVF | Human Reproduction. 2014; 29(2):293-302 | Study purpose: Not related to QoL/treatment satisfaction |
| Lykeridou, K. et al, 2009 | The impact of infertility diagnosis on psychological status of women undergoing fertility treatment | Journal of Reproductive and Infant Psychology. 2009; 27(3):223-237 | Setting not treatment-specific |
| Makanjuola, A. B. et al, 2010 | Predictive factors for psychiatric morbidity among women with infertility attending a gynaecology clinic in Nigeria | African Journal of Psychiatry. 2010; 13(1):36-42 | Setting not treatment-specific |
| Maria, Q. R. et al, 2010 | Descriptive study on factors modulating psychosocial condition of infertile couples | Journal of Psychosomatic Obstetrics and Gynecology. 2010; 31():119 | Setting not treatment-specific |
| Martins, M. V. et al, 2012 | Infertility disclosure moderates the relationship between social support and fertility stress in patients following unsuccessful treatments | Human Reproduction. 2012; 27(Suppl2):ii98-ii100 | Study purpose: Not related to QoL/treatment satisfaction |
| Masoumi, S. et al, 2013 | Quality of life in infertile couples in Fatemiyeh Hospital in Hamedan | Iranian Journal of Reproductive Medicine. 2013; 11():104 | Setting not treatment-specific |
| Melo, C. et al, 2012 | Does the FertiQoL assess quality of life? Results from the validation of the Portuguese version of the FertiQoL | Human Reproduction. 2012; 27(Suppl2):ii268-ii273 | Study purpose: PRO development methodology paper, no results reported |
| Mohammad Alizadeh Charandabi, S. et al, 2012 | Health-Related Quality of Life and its Predictive Factors among Infertile Women | Journal of Caring Sciences. 2012; 1(3):159-64 | Setting not treatment-specific |
| Monga, M., 2004 | Impact of infertility on quality of life, marital adjustment, and sexual function | Urology. 2004; 63(1):126-130 | Setting not treatment-specific |
| Moreno-Rosset, C. et al, 2009 | [Validation of the Questionnaire of Emotional Maladjustment and Adaptive Resources in Infertility (DERA)] | Psicothema. 2009; 21(1):118-23 | Study purpose: Not related to QoL/treatment satisfaction |
| Moura-Ramos, M. et al, 2012 | Assessing infertility stress: Re-examining the factor structure of the Fertility Problem Inventory | Human Reproduction. 2012; 27(2):496-502 | Study purpose: PRO development methodology paper, no results reported |
| Najafi, M. et al, 2015 | The effectiveness of emotionally focused therapy on enhancing marital adjustment and quality of life among infertile couples with marital conflicts | International Journal of Fertility and Sterility. 2015; 9(2):238-246 | Setting not treatment-specific |
| Najafi, M. et al, 2015 | The study of the effectiveness of couple emotionally focused therapy (EFT) on increasing marital adjustment and improving the physical and psychological health of the infertile couples. [Persian] | Iranian Journal of Obstetrics, Gynecology and Infertility. 2015; 17(133):8-21 | Setting not treatment-specific |
| Nehir, A. et al, 2012 | Evaluation of psychosocial and sexual profiles of the infertile couples | Human Reproduction. 2012; 27(Suppl2):ii268-ii273 | Setting not treatment-specific |
| Nelen, W. L. D. M. et al, 2010 | Measuring patient centredness, the neglected outcome measure in fertility care | Human Reproduction. 2010; 25():i250-i251 | Study purpose: Not related to QoL/treatment satisfaction |
| Nelson, C. J. et al, 2008 | Prevalence and predictors of sexual problems, relationship stress, and depression in female partners of infertile couples | Journal of Sexual Medicine. 2008; 5(8):1907-1914 | Setting not treatment-specific |
| Newton, C. R. et al, 1999 | The fertility problem inventory: Measuring perceived infertility- related stress | Fertility and Sterility. 1999; 72(1):54-62 | Study purpose: PRO development methodology paper, no results reported |
| Noorbala, A. A. et al, 2009 | Psychiatric disorders among infertile and fertile women | Social Psychiatry and Psychiatric Epidemiology. 2009; 44(7):587-591 | Setting not treatment-specific |
| Nourani, S. et al, 2012 | Comparison of quality of life in fertile and infertile women referred to the public clinics in Mashhad | Iranian Journal of Obstetrics, Gynecology and Infertility. 2012; 15(7):24-31 | Setting not treatment-specific |
| Ockhuijsen, H. D. L. et al, 2013 | The PRCI study: Design of a randomized clinical trial to evaluate a coping intervention for medical waiting periods used by women undergoing a fertility treatment | BMC women's health. 2013; 13(1):35 | Protocol only: No study data |
| Onat, G. et al, 2012 | Effects of infertility on gender differences in marital relationship and quality of life: A case-control study of Turkish couples | European Journal of Obstetrics Gynecology and Reproductive Biology. 2012; 165(2):243-248 | Setting not treatment-specific |
| Oron, G. et al, 2015 | A prospective study using Hatha Yoga for stress reduction among women waiting for IVF treatment | Reproductive BioMedicine Online. 2015; 30(5):542-548 | Setting not treatment-specific |
| Pakpour, A. H. et al, 2012 | Prevalence and risk factors of the female sexual dysfunction in a sample of infertile Iranian women | Archives of Gynecology and Obstetrics. 2012; 286(6):1589-1596 | Setting not treatment-specific |
| Patel, M, 2013 | Health related quality of life in women and their partners accessing infertility care at an urban, public tertiary referral centre in South Africa | Human Reproduction. 2013; 28():i138 | Setting not treatment-specific |
| Pedro, J. et al, 2013 | Positive experiences of patient-centred care are associated with intentions to comply with fertility treatment: Findings from the validation of the Portuguese version of the PCQ-Infertility tool | Human Reproduction. 2013; 28(9):2462-2472 | Study purpose: Not related to QoL/treatment satisfaction |
| Pellicano, M. et al, 2003 | Carbon dioxide versus normal saline as a uterine distension medium for diagnostic vaginoscopic hysteroscopy in infertile patients: a prospective, randomized, multicenter study | Fertility & Sterility. 2003; 79(2):418-21 | Study purpose: Not related to QoL/treatment satisfaction |
| Peng, T. et al, 2011 | Testing the psychometric properties of Mandarin version of the fertility problem inventory (M-FPI) in an infertile Chinese sample | Journal of Psychosomatic Obstetrics and Gynecology. 2011; 32(4):173-181 | Study purpose: PRO development methodology paper, no results reported |
| Peterson, B. et al, 2009 | Treating infertility stress in patients undergoing in vitro fertilization (IVF) using acceptance and commitment therapy (ACT) | Fertility and Sterility. 2009; 1():S18 | Study design: Single case study only |
| Peterson, B. et al, 2013 | A typology of coping in couples undergoing infertility treatment | Human Reproduction. 2013; 28():i280 | Setting not treatment-specific |
| Peterson, B. et al, 2014 | Are severe depressive symptoms associated with infertility-related distress in individuals and their partners? | Human Reproduction. 2014; 29(1):76-82 | Setting not treatment-specific |
| Pinar, G. et al, 2012 | Quality of life, anxiety and depression in Turkish women prior to receiving assisted reproductive techniques | International Journal of Fertility and Sterility. 2012; 6(1):1-12 | Setting not treatment-specific |
| Poddar, S. et al, 2014 | Psychological profile of women with infertility: A comparative study | Industrial Psychiatry Journal. 2014; 23(2):117-26 | Setting not treatment-specific |
| Pook, M. et al, 2002 | A questionnaire assessing infertility distress in andrological patients. [German] | Reproduktionsmedizin. 2002; 18(6):327-332 | Study purpose: PRO development methodology paper, no results reported |
| Quant, H. S. et al, 2013 | Reproductive implications of psychological distress for couples undergoing IVF | Journal of Assisted Reproduction and Genetics. 2013; 30(11):1451-1458 | Setting not treatment-specific |
| Radwan, D. N. et al, 2009 | Comorbidity between depression, anxiety disorders and infertility: Effect on quality of life in Egyptian women | European Neuropsychopharmacology. 2009; 19():S373 | Setting not treatment-specific |
| Ragni, G. et al, 2005 | Health-related quality of life and need for IVF in 1000 Italian infertile couples | Human Reproduction. 2005; 20(5):1286-1291 | Setting not treatment-specific |
| Ramezanzadeh, F. et al, 2004 | A survey of relationship between anxiety, depression and duration of infertility | BMC Women's Health. 2004; 4(9): | Setting not treatment-specific |
| Rashidi, B. et al, 2008 | Health-related quality of life in infertile couples receiving IVF or ICSI treatment | BMC Health Services Research. 2008; 8():186 | Setting not treatment-specific |
| Ried, K. et al, 2013 | Quality of life, coping strategies and support needs of women seeking Traditional Chinese Medicine for infertility and viable pregnancy in Australia: A mixed methods approach | BMC Women's Health. 2013; 13(1):17 | Setting not treatment-specific |
| Roca de Bes, M. et al, 2013 | Developing and testing a new instrument to measure women's satisfaction with controlled ovarian stimulation treatment | Journal of Psychosomatic Obstetrics & Gynecology. 2013; 34(1):53-8 | Study purpose: PRO development methodology paper, no results reported |
| Romera, N. et al, 2010 | Assessment quality of life in cross-border patients using the new tool "fertiqol" | Human Reproduction. 2010; 25():i77-i78 | Setting not treatment-specific |
| Sadeghian, E. et al, 2012 | Psychiatric problems in infertile women referring to infertility clinic of Hamadan Fatemiyeh hospital | International Journal of Fertility and Sterility. 2012; 6():146-147 | Setting not treatment-specific |
| Saito, Y. et al, 2009 | Trial development of the Cognitive Appraisal Scale for Infertility (CASI) (version 1) | Fertility and Sterility. 2009; 91(6):2596-2601 | Study purpose: PRO development methodology paper, no results reported |
| Salomao, P. et al, 2015 | Sexual function of women under treatment for infertility | Journal of Sexual Medicine. 2015; 12():60 | Setting not treatment-specific |
| Sanders, K. A. et al, 1999 | Psychosocial stress and treatment outcome following assisted reproductive technology | Human Reproduction. 1999; 14(6):1656-1662 | Setting not treatment-specific |
| Schanz, S. et al, 2005 | A new quality-of-life measure for men experiencing involuntary childlessness | Human Reproduction. 2005; 20(10):2858-2865 | Study purpose: PRO development methodology paper, no results reported |
| Schmid, J. et al, 2004 | Infertility caused by PCOS - Health-related quality of life among Austrian and Moslem immigrant women in Austria | Human Reproduction. 2004; 19(10):2251-2257 | Population: Not infertility |
| Sejbaek, C. S. et al, 2009 | The impact of social relations on incident severe depressive symptoms among couples in unsuccessful fertility treatment | Molecular Human Reproduction. 2009; 24(Suppl1):i91-i92 | Setting not treatment-specific |
| Sexton, M. B. et al, 2010 | Measuring resilience in women experiencing infertility using the CD-RISC: Examining infertility-related stress, general distress, and coping styles | Journal of Psychiatric Research. 2010; 44(4):236-241 | Setting not treatment-specific |
| Sezgin, H. et al, 2012 | The effect of disability and psychiatric symptoms on the quality of life in among infertility patients, Infertilite nedeni ile basvuran hastalarda ruhsal belirtiler ve yetiyitiminin yasam kalitesine etkisi. [Turkish, English] | Klinik Psikofarmakoloji Bulteni. 2012; 22():S125 | Setting not treatment-specific |
| Shahid, S. et al, 2009 | Depression in infertile couples | Journal of the College of Physicians and Surgeons--Pakistan : JCPSP. 2009; 19(6):395-396 | Setting not treatment-specific |
| Shindel, A. W. et al, 2008 | Sexual Function and Quality of Life in the Male Partner of Infertile Couples: Prevalence and Correlates of Dysfunction | Journal of Urology. 2008; 179(3):1056-1059 | Setting not treatment-specific |
| Smith, C. et al, 2010 | The effect of acupuncture on psychosocial outcomes for women experiencing infertility: Findings from a pilot pragmatic randomised controlled trial | Australian and New Zealand Journal of Obstetrics and Gynaecology. 2010; 50():9-10 | Setting not treatment-specific |
| Sreshthaputra, O. et al, 2008 | Gender differences in infertility-related stress and the relationship between stress and social support in Thai infertile couples | Journal of the Medical Association of Thailand. 2008; 91(12):1769-73 | Setting not treatment-specific |
| Stadnicka, G. et al, 2014 | The sexuality of women treated for infertility | European Journal of Contraception and Reproductive Health Care. 2014; 19():S225-S226 | Setting not treatment-specific |
| Stenyaeva, N. et al, 2015 | Open cross-sectional study of the clinical types of sexual dysfunction, personality traits and psychological status in women with infertility | Journal of Sexual Medicine. 2015; 12():3-4 | Setting not treatment-specific |
| Stewart, D. E. et al, 1992 | A prospective study of the effectiveness of brief professionally-led support groups for infertility patients | International Journal of Psychiatry in Medicine. 1992; 22(2):173-182 | Setting not treatment-specific |
| Sun, Z. G. et al, 2012 | [Effects of acupuncture combined Chinese materia medica for tonifying shen and soothing gan on the anxiety and depression of patients with in vitro fertilization and embryo transplantation and on the treatment outcomes]. [Chinese] | Zhongguo Zhong xi yi jie he za zhi Zhongguo Zhongxiyi jiehe zazhi = Chinese journal of integrated traditional and Western medicine / Zhongguo Zhong xi yi jie he xue hui, Zhongguo Zhong yi yan jiu yuan zhu ban. 2012; 32(8):1023-1027 | Setting not treatment-specific |
| Sutton, C. et al, 2015 | A comparison of stress levels in women undergoing single versus multiple acupuncture session prior to embryo transfer | Fertility and Sterility. 2015; 1)():e36-e37 | Setting not treatment-specific |
| Tarabusi, M. et al, 2004 | Psychological group support attenuates distress of waiting in couples scheduled for assisted reproduction | Journal of Psychosomatic Obstetrics and Gynecology. 2004; 25(3-4):273-279 | Setting not treatment-specific |
| Teskereci, G. et al, 2013 | Effect of lifestyle on quality of life of couples receiving infertility treatment | Journal of Sex & Marital Therapy. 2013; 39(6):476-92 | Setting not treatment-specific |
| Timur Tashan, S. et al, 2013 | Traditional practices used by the infertile women to become pregnant and their effects on the quality of life | International Journal of Nursing Practice. 2013; 19(5):516-522 | Setting not treatment-specific |
| Turner, K. et al, 2013 | Stress reduction during in vitro fertilization | Fertility and Sterility. 2013; 1():S25-S26 | Setting not treatment-specific |
| Ugur, A. S. et al, 2014 | The effect of perceived quality of life on physical, emotional, social, relational situations in women with fertility problems | Fertility and Sterility. 2014; 1():e312 | Setting not treatment-specific |
| Valsangkar, S. et al, 2011 | An evaluation of the effect of infertility on marital, sexual satisfaction indices and health-related quality of life in women | Journal of Human Reproductive Sciences. 2011; 4(2):80-85 | Setting not treatment-specific |
| Van Den Broeck, U. E. et al, 2010 | Psychosocial determinants of the decision making process in continuing or discontinuing in-vitro-fertilisation treatment: A psychological analysis | Journal of Reproductive and Infant Psychology. 2010; 28 (3)():e16 | Setting not treatment-specific |
| Van Dongen, A. J. C. et al, 2011 | Feasibility of screening patients starting IVF for risk at emotional problems: A process evaluation | Human Reproduction. 2011; 26():i269 | Study purpose: Not related to QoL/treatment satisfaction |
| Van Dongen, A. J. C. et al, 2012 | Feasibility of screening patients for emotional risk factors before in vitro fertilization in daily clinical practice: a process evaluation | Human Reproduction. 2012; 27(12):3493-501 | Setting not treatment-specific |
| Van Dongen, A. J. C. et al, 2013 | Psychosocial predictors of dropout in in vitro fertilization | Human Reproduction. 2013; 28():i267 | Study purpose: Not related to QoL/treatment satisfaction |
| Van Balen, F. et al, 1994 | Factors influencing the well-being of long-term infertile couples | Journal of Psychosomatic Obstetrics and Gynaecology. 1994; 15(3):157-164 | Setting not treatment-specific |
| Van Den Broeck, U. E. et al, 2009 | Predictors of intrusiveness of the infertility experience in patients starting IVF-treatment | Fertility and Sterility. 2009; 1():S33 | Setting not treatment-specific |
| Van Den Broeck, U. E. et al, 2009 | Infertility specific concerns versus more general psychological and personality characteristics in predicting psychological distress in starting IVF | Molecular Human Reproduction. 2009; 24(Suppl1):i93 | Setting not treatment-specific |
| Van Den Broeck, U. E. et al, 2010 | Predictors of psychological distress in patients starting IVF treatment: Infertility-specific versus general psychological characteristics | Human Reproduction. 2010; 25(6):1471-1480 | Setting not treatment-specific |
| Veloso-Martins, M. et al, 2010 | The effects of perceived social support and coping strategies on infertility stress in women: Testing for mediation using structural equation modelling | Human Reproduction. 2010; 25():i280 | Setting not treatment-specific |
| Vislava, G. V. et al, 2010 | Psychic well-being and quality of life of women conceiving through in vitro fertilization | Journal of Psychosomatic Obstetrics and Gynecology. 2010; 31():79 | Study purpose: Postnatal QoL |
| Wang, K. et al, 2007 | Psychological characteristics and marital quality of infertile women registered for in vitro fertilization-intracytoplasmic sperm injection in China | Fertility and Sterility. 2007; 87(4):792-798 | Setting not treatment-specific |
| Weedin, E. A. et al, 2014 | Initiating infertility treatment: Does it improve or worsen anxiety and/or depression? | Fertility and Sterility. 2014; 1)():e13-e14 | Setting not treatment-specific |
| Whitlow, N. R. et al, 2011 | The patient health questionnaire (PHQ) is a poor psychological screening tool in in vitro fertilization (IVF) patients | Fertility and Sterility. 2011; 1)():S11 | Setting not treatment-specific |
| Wischmann, T. et al, 2001 | Psychosocial characteristics of infertile couples: A study by the 'Heidelberg Fertility Consultation Service' | Human Reproduction. 2001; 16(8):1753-1761 | Setting not treatment-specific |
| Wischmann, T. et al, 2009 | Psychosocial characteristics of women and men attending infertility counselling | Human Reproduction. 2009; 24(2):378-85 | Setting not treatment-specific |
| Wischmann, T. et al, 2012 | A 10-year follow-up study of psychosocial factors affecting couples after infertility treatment | Human Reproduction. 2012; 27(11):3226-3232 | Study purpose: Postnatal QoL |
| Wischmann, T. et al, 2014 | Sexuality, self-esteem and partnership quality in infertile women and men | Geburtshilfe und Frauenheilkunde. 2014; 74(8):759-763 | Study purpose: Not related to QoL/treatment satisfaction |
| Wruble, A. W. et al, 2012 | Women in fertility treatments: Adjustment, marital satisfaction, and sexual quality of life | Journal of Sexual Medicine. 2012; 9():162-163 | Setting not treatment-specific |
| Zamani, S. N. et al, 2013 | Comparison of depression and life quality of fertile and infertile women and those with frequent abortions. [Persian] | Journal of Babol University of Medical Sciences. 2013; 15(6):78-83 | Setting not treatment-specific |
