# Supplementary material for: A review of patient-reported outcome measures to assess female infertility-related quality of life
Source: Health Qual Life Outcomes. 2017 Apr 27;15:86. doi: 10.1186/s12955-017-0666-0 (PMC5408488; doi:10.1186/s12955-017-0666-0)
Supplement: Supplementary file 5 — PROs excluded from detailed analysis. Table listing each identified PRO excluded from detailed analysis, detailing each PRO acronym, PRO name, no. studies identified as using the PRO, and the reason for exclusion. (DOCX 55 kb) [file 12955_2017_666_MOESM5_ESM.docx]

### PRO measures excluded from detailed analysis

Table S9: PROs excluded from detailed analysis, n=73

| PRO Acronym | PRO Name | No. Studies using PRO | Reason for Exclusion |
| --- | --- | --- | --- |
| ASWB | Amsterdam Scale of Well-Being | 1 | Is not infertility specific |
| - | Asazawa 2015 Unnamed PRO | 1 | Has not been used by at least two separate research groups |
| AAI | Attitudes About Infertility | 1 | Has not been used by at least two separate research groups |
| BAI | Beck Anxiety Inventory | 1 | Is not infertility specific |
| BDI | Beck Depression Inventory | 19 | Is not infertility specific |
| - | Beutel 1999 Unnamed PRO | 1 | Has not been used by at least two separate research groups |
| - | Bitzer 2011 Unnamed PRO | 1 | Has not been used by at least two separate research groups |
| - | Bladh Blomquist 2015 Unnamed PRO | 1 | Has not been used by at least two separate research groups |
| BABS | Bradbern Affect Balance Scales | 1 | Is not infertility specific |
| BSI | Brief Symptom Inventory | 4 | Is not infertility specific |
| - | Butler 2005 Unnamed PRO | 1 | Has not been used by at least two separate research groups |
| CPI | California Psychological Inventory | 1 | Is not infertility specific |
| CES-D | Center for Epidemiologic Studies Scale -– Depression | 6 | Is not infertility specific |
| CART | Concerns During Assisted Reproductive Technology | 1 | Has not been used by at least two separate research groups |
| COSI | Controlled Ovarian Stimulation Impact Measure | 1 | Has not been used by at least two separate research groups |
| CMI | Cornell Medical Index | 1 | Is not infertility specific |
| DRK | Daily Record Keeping | 6 | Psychometric publications are not available |
| DACL | Depression Adjective Checklist | 3 | Is not infertility specific |
| DEQ | Depressive Experiences Questionnaire | 1 | Is not infertility specific |
| EI-14 | Effects of Infertility - 14 item | 2 | Has not been used by at least two separate research groups |
| EI-7 | Effects of Infertility – 7 item | 1 | Has not been used by at least two separate research groups |
| EHIQ | Emotional Health in Infertility Questionnaire | 1 | Has not been used by at least two separate research groups |
| FAI | Feelings About Infertility Questionnaire | 1 | Psychometric publications are not available |
| FTI | Feelings Toward Infertility | 1 | Has not been used by at least two separate research groups |
| FertiMed | FertiMed | 1 | Has not been used by at least two separate research groups |
| FERTSQ | Frozen Embryo Replacement Treatment Satisfaction Questionnaire | 1 | Has not been used by at least two separate research groups |
| GHQ | General Health Questionnaire | 5 | Is not infertility specific |
| HAM-D | Hamilton Rating Scale for Depression | 1 | Is not infertility specific |
| HADS | Hospital Anxiety and Depression Scale | 8 | Is not infertility specific |
| - | Hynes 1992 Unnamed PRO | 1 | Has not been used by at least two separate research groups |
| IES | Impact of Events Scale | 1 | Is not infertility specific |
| ISS | Infertility and Strain Scale | 2 | Has not been used by at least two separate research groups |
| IDQ | Infertility Difficulties Questionnaire | 1 | Has not been used by at least two separate research groups |
| IRS | Infertility Reaction Scale | 2 | Psychometric publications are not available |
| IBS | Infertility Specific Distress | 1 | Has not been used by at least two separate research groups |
| ISWD-SF | Infertility Specific Well-Being and Distress Scales - Short Form | 2 | Has not been used by at least two separate research groups |
| IDATE | Inventaria de Ansiedade Traco- Estado | 1 | Is not infertility specific |
| IPAT-Anxiety | IPAT Anxiety Scale Questionnaire | 1 | Is not infertility specific |
| IVF-AS | IVF Attitude Scale | 1 | Has not been used by at least two separate research groups |
| IVF-SI | IVF Stress Inventory | 1 | Has not been used by at least two separate research groups |
| - | Kettel 2004 Unnamed PRO | 1 | Has not been used by at least two separate research groups |
| - | Leiblum 1987 Unnamed PRO | 1 | Has not been used by at least two separate research groups |
| - | Letur-Kornisch 2001 Unnamed PRO | 1 | Has not been used by at least two separate research groups |
| - | Mahlstedt 1987 Unnamed PRO | 1 | Has not been used by at least two separate research groups |
| MAS | Manifest Anxiety Scale | 1 | Is not infertility specific |
| MAACL | Mean Affect Adjective Check List | 1 | Is not infertility specific |
| MHI-5 | Mental Health Inventory | 1 | Is not infertility specific |
| MMPI | Minnesota Multiphasic Inventory | 1 | Is not infertility specific |
| - | Nasseri 2000 Unnamed PRO | 1 | Has not been used by at least two separate research groups |
| - | Newman 1991 Unnamed PRO | 1 | Has not been used by at least two separate research groups |
| - | Pang 2003 Unnamed PRO | 1 | Has not been used by at least two separate research groups |
| PSS | Perceived Stress Scale | 2 | Is not infertility specific |
| PANAS | Positive and Negative Affect Schedule | 3 | Is not infertility specific |
| POMS | Profile of Mood States | 8 | Is not infertility specific |
| PET-ART | Psychological Evaluation Test After the Use of Assisted Reproductive Techniques | 1 | Has not been used by at least two separate research groups |
| PGWB | Psychological General Well-Being Index | 1 | Is not infertility specific |
| - | Reading 1998 Unnamed PRO | 1 | Has not been used by at least two separate research groups |
| RSES | Rosenberg Self-Esteem Scale | 1 | Is not infertility specific |
| ScreenIVF | SCREENIVF | 1 | Has not been used by at least two separate research groups |
| - | Sedbon 2006 Unnamed PRO | 1 | Has not been used by at least two separate research groups |
| SRSS | Self-Rated Stress Scale | 1 | Is not infertility specific |
| SAS | Self-Rating Anxiety Scale | 1 | Is not infertility specific |
| SF-36 | Short Form Health Survey 36 | 1 | Is not infertility specific |
| - | Solnica 2009 Unnamed PRO | 1 | Has not been used by at least two separate research groups |
| STAI | State-Trait Anxiety Inventory | 39 | Is not infertility specific |
| SCL-90-R | Symptom Checklist 90 | 3 | Is not infertility specific |
| - | Utsunomiya 2012 Unnamed PRO | 1 | Has not been used by at least two separate research groups |
| VENUsf | VENUsf 14-item | 1 | Has not been used by at least two separate research groups |
| VZDS | Von Zerssen Depression Scale | 1 | Is not infertility specific |
| TEPE | Waiting Period Evaluation Test | 1 | Is not infertility specific |
| - | Woodward 2015 Unnamed PRO | 1 | Has not been used by at least two separate research groups |
| WHOQOL | World Health Organisation Quality of Life | 3 | Is not infertility specific |
| SDS | Zung Self-rating Depression Scale | 1 | Is not infertility specific |
